# Quality care during labour and birth: a multi-country analysis of health system bottlenecks and potential solutions

**DOI:** 10.1186/1471-2393-15-S2-S2

**Published:** 2015-09-11

**Authors:** Gaurav Sharma, Matthews Mathai, Kim E Dickson, Andrew Weeks, G Justus Hofmeyr, Tina Lavender, Louise Tina Day, Jiji Elizabeth Mathews, Sue Fawcus, Aline Simen-Kapeu, Luc de Bernis

**Affiliations:** 1 Maternal, Adolescent, Reproductive and Child Health (MARCH) Centre, London School of Hygiene and Tropical Medicine, London, WC1E 7HT, United Kingdom; 2 Department of Infectious Disease Epidemiology, London School of Hygiene and Tropical Medicine, London, WC1E 7HT, UK; 3 Department of Maternal, Newborn, Child and Adolescent Health, World Health Organization, 20 Avenue Appia, 1211 Geneva 27, Switzerland; 4 Health Section, Programme Division, UNICEF Headquarters, 3 United Nations Plaza, New York, 10017, USA; 5 Sanyu Research Unit, University of Liverpool, c/o Liverpool Women's Hospital, Crown Street, Liverpool, L8 7SS, UK; 6 Department of Obstetrics and Gynaecology, East London Hospital Complex, University of the Witwatersrand, University of Fort Hare, Eastern Cape Department of Health, East London, South Africa; 7 University of Manchester School of Nursing, Midwifery & Social Work, Jean McFarlane Building University Place, Oxford Road, Manchester, M13 9PL, UK; 8 LAMB, Integrated Rural Health & Development, Dinajpur, 5250, Bangladesh; 9 Department of Obstetrics and Gynaecology, Christian Medical College and Hospital, Vellore, Tamil Nadu, India; 10 Department of Obstetrics & Gynaecology, University of Cape Town, Observatory 7925, Cape Town, South Africa; 11 UN Population Fund, Geneva, Switzerland

## Abstract

**Background:**

Good outcomes during pregnancy and childbirth are related to availability, utilisation and effective implementation of essential interventions for labour and childbirth. The majority of the estimated 289,000 maternal deaths, 2.8 million neonatal deaths and 2.6 million stillbirths every year could be prevented by improving access to and scaling up quality care during labour and birth.

**Methods:**

The bottleneck analysis tool was applied in 12 countries in Africa and Asia as part of the *Every Newborn *Action Plan process. Country workshops engaged technical experts to complete the survey tool, which is designed to synthesise and grade health system "bottlenecks", factors that hinder the scale up, of maternal-newborn intervention packages. We used quantitative and qualitative methods to analyse the bottleneck data, combined with literature review, to present priority bottlenecks and actions relevant to different health system building blocks for skilled birth attendance and basic and comprehensive emergency obstetric care.

**Results:**

Across 12 countries the most critical bottlenecks identified by workshop participants for skilled birth attendance were health financing (10 out of 12 countries) and health workforce (9 out of 12 countries). Health service delivery bottlenecks were found to be the most critical for both basic and comprehensive emergency obstetric care (9 out of 12 countries); health financing was identified as having critical bottlenecks for comprehensive emergency obstetric care (9 out of 12 countries). Solutions to address health financing bottlenecks included strengthening national financing mechanisms and removing financial barriers to care seeking. For addressing health workforce bottlenecks, improved human resource planning is needed, including task shifting and improving training quality. For health service delivery, proposed solutions included improving quality of care and establishing public private partnerships.

**Conclusions:**

Progress towards the 2030 targets for ending preventable maternal and newborn deaths is dependent on improving quality of care during birth and the immediate postnatal period. Strengthening national health systems to improve maternal and newborn health, as a cornerstone of universal health coverage, will only be possible by addressing specific health system bottlenecks during labour and birth, including those within health workforce, health financing and health service delivery.

## Background

Improvements in maternal and newborn health have been important global priorities over the past decade. Pregnancy and perinatal outcomes are closely linked to health, nutritional and educational outcomes of the child [1]. Achieving Millennium Development Goal targets for maternal and child survival are an integral part of the UN Secretary General's Global Strategy for Women's and Children's Health [2]. Despite substantial declines in maternal deaths (decline of 45% from 1990 levels) and increasing rates of facility deliveries, estimates indicate that 289,000 maternal deaths [3], 2.8 million neonatal deaths [4] and 2.65 million stillbirths occur annually [5]. As the majority of these deaths occur during labour, childbirth and the early postnatal period [5,6], there are limited alternatives to the provision of high quality professional care at facilities especially in low and middle income countries (LMICs). Ending preventable maternal deaths, neonatal deaths and stillbirths is possible given declining worldwide trends [7], widespread political support and focused action in countries [2]. Discussions to date have set the global target for ending preventable maternal mortality at <70 maternal deaths per 100,000 live births by 2030, and with no country having MMR of >140 deaths per 100,000 live births by 2030 [7]. For newborn mortality, the global targets are to achieve NMR of 7 per 1000 live births by 2035 with NMR of 10 or less in countries [8]. Similarly, for stillbirths, national targets are to achieve 10 or less stillbirths per 1000 total births by 2035 which corresponds to a global average of 8 stillbirths per 1000 total births [8]. These targets will only be met by strengthening existing health systems in countries and improving intrapartum and postnatal quality of care.

An increased focus on quality care at the time of birth has quadruple returns on investment through the reduction of maternal and neonatal deaths, prevention of stillbirths and future disability [9]. Recent estimates indicate that closure of the quality gap through the provision of effective care for all women and newborn babies delivering in facilities could prevent an estimated 113,000 maternal deaths, 531,000 stillbirths, and 1·325 million neonatal deaths annually by 2020 [10].

**Figure 1:**
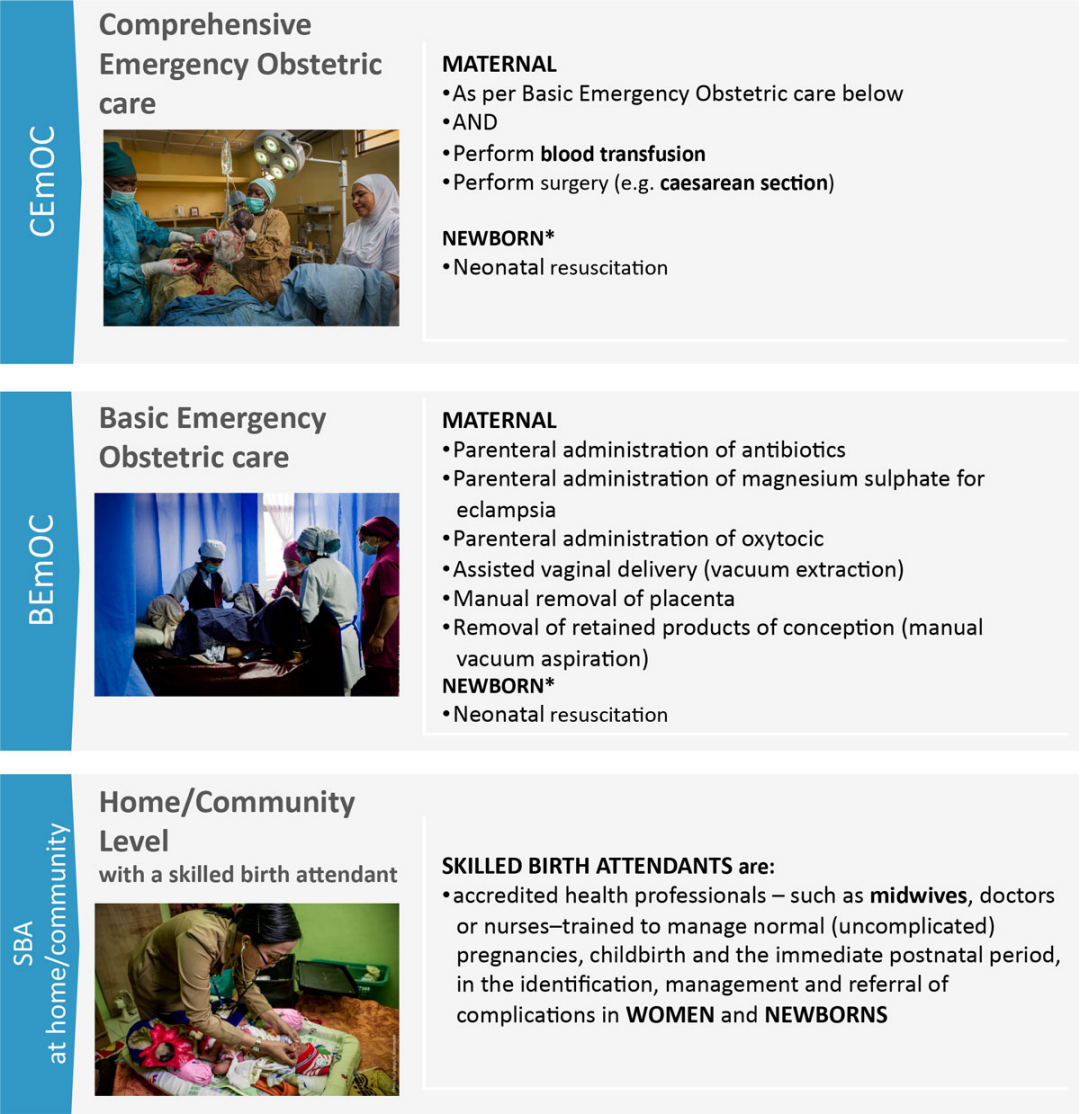
**Labour and birth packages by level of care**. * Ongoing process to define newborn care interventions by level of care. Comprehensive emergency obstetric care image source: Karen Kasmauski/MCSP. Basic emergency obstetric care image source: K. Holt/Jhpiego. Home/community level image source: K. Kasmauski/Jhpiego.

Traditionally, programmes to improve maternal and newborn health have largely focussed on increasing coverage of births by skilled birth attendants. The most efficient way of achieving increased coverage is through provision of care by skilled teams at appropriate maternity facilities that have the capacity to provide 24/7 care for normal labour and childbirth, and manage or refer any complications that may arise during labour, childbirth and the immediate post-natal period [11]. Provision of a seamless, high-quality maternity care pathway at facilities requires a multi-dimensional approach. Communities need to be empowered to demand high-quality services including well-functioning referral and transport mechanisms. Skilled and motivated teams should be available at facilities equipped with necessary medicines and commodities, working in enabling environments that promote evidence based practices and client-centred, respectful maternity care services. Furthermore, robust facility management and administrative systems with in-built accountability mechanisms are needed. Consensus exists on a minimum care package of interventions required during pregnancy and childbirth [12]. In addition to this package for routine care, some women and babies may require higher-level care for complications. Facilities that provide such emergency obstetric and neonatal care are classified as Basic Emergency Obstetric Care (BEmOC) facilities or Comprehensive Emergency Obstetric Care (CEmOC) facilities (Figure 1) based on the provision of specified signal functions [13].

This paper is part of a series on quality maternal and newborn care; it analyses bottlenecks and solutions specific to the provision of skilled care at birth, basic and comprehensive emergency obstetric care. Given the current status of maternal and newborn health and the momentum gathering around ending preventable maternal and neonatal deaths through improved quality of care during childbirth and the immediate postnatal period, the objectives of this paper are to:

1. Use a 12 country analysis to explore health system bottlenecks affecting the scale-up of quality care during labour, childbirth and immediate postnatal period

2. Present the solutions to overcome the most significant bottlenecks including learning from the 12-country analyses, literature review and programme experience

3. Discuss policy and programmatic implications and propose priority actions for programme scale up.

## Methods

This study used quantitative and qualitative research methods to collect information, assess health system bottlenecks and identify solutions to scale up maternal and newborn care interventions in 12 high burden countries: Afghanistan, Cameroon, Democratic Republic of Congo (DRC), Kenya, Malawi, Nigeria, Uganda, Bangladesh, India, Nepal, Pakistan and Vietnam.

### Data Collection

The maternal-newborn bottleneck analysis tool (see Additional file 1) was developed to assist countries in the identification of bottlenecks to the scale up and provision of nine maternal and newborn health interventions across the seven health system building blocks as described previously [14,15]. The tool (see Additional file 1) was utilised during a series of national consultations supported by the global *Every Newborn *Steering Group between July 1st and December 31st, 2013. The workshops for each country included participants from Ministries of Health, UN agencies, the private sector, non-governmental organisations (NGOs), professional associations, academia, bilateral agencies and other stakeholders. For each workshop, a facilitator, orientated on the tool, facilitated the discussions and helped groups reach consensus on specific bottlenecks for health system building blocks [15]. This paper, second in the series, focuses on the bottlenecks related to scale up of SBA, BEmOC and CEmOC.

Tracer interventions were defined for each package to focus the workshop discussion. For skilled care at birth, the tracer intervention was the use of the partograph. The partograph is usually available as a pre-printed paper form on which observations on the mother and foetus during labour are recorded. The aim of the partograph is to provide a pictorial overview of labour to alert skilled birth attendants to deviations in maternal or foetal wellbeing and labour progress. For BEmOC, the tracer intervention was assisted vaginal delivery, which refers to the application of either forceps or a vacuum device to assist the mother in effecting vaginal delivery of a foetus. For CEmOC, the tracer intervention was caesarean section, the procedure of delivering a baby through incisions made in the mother's abdominal wall and uterus.

### Data analysis methods

**Figure 2:**
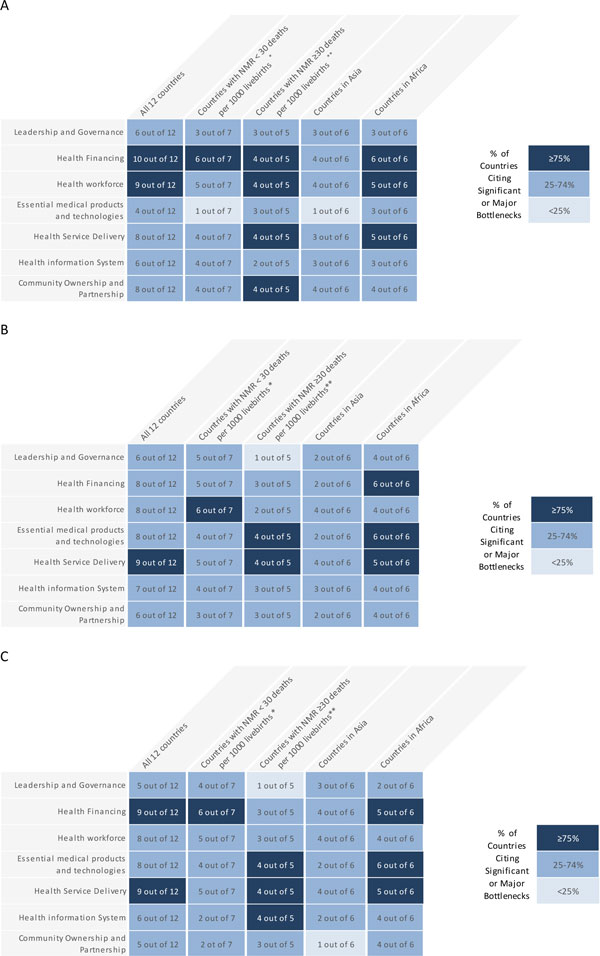
**Very major or significant health system bottlenecks for labour and birth**. NMR: Neonatal Mortality Rate *Cameroon, Kenya, Malawi, Uganda, Bangladesh, Nepal, Vietnam. **Democratic Republic of Congo, Nigeria, Afghanistan, India, Pakistan. See additional file 2 for more details. Part A: Grading according to very major or significant health system bottlenecks for skilled birth attendance as reported by twelve countries combined. Part B: Grading according to very major or significant health system bottlenecks for basic emergency obstetric care (BEmOC) as reported by twelve countries combined. Part C: Grading according to very major or significant health system bottlenecks for comprehensive emergency obstetric care (CEmOC) as reported by twelve countries combined.

**Figure 3:**
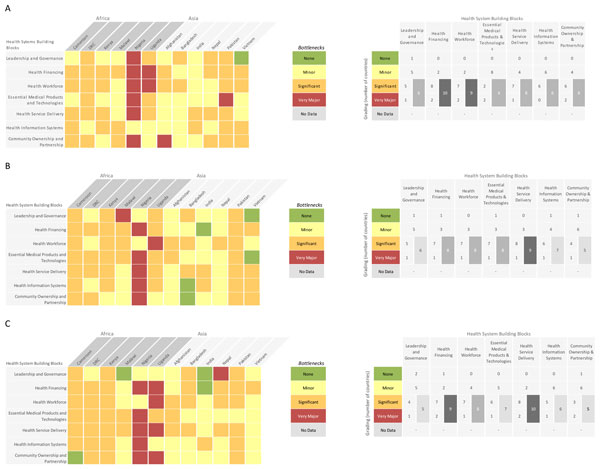
**Individual country grading of health system bottlenecks for labour and birth**. Part A: Heat map showing individual country grading of health system bottlenecks for skilled birth attendance (SBA) and table showing total number of countries grading significant or major bottleneck for calculating priority building blocks. Part B: Heat map showing individual country grading of health system bottlenecks for basic emergency obstetric care (BEmOC) and table showing total number of countries grading significant or major bottleneck for calculating priority building blocks. Part C: Heat map showing individual country grading of health system bottlenecks for comprehensive emergency obstetric care (CEmOC) and table showing total number of countries grading significant or major bottleneck for calculating priority building blocks. DRC: Democratic Republic of the Congo.

Data received from each country were analysed and the graded health system building blocks were converted into heat maps. Bottlenecks for each health system building block were graded using one of the following options: not a bottleneck (=1), minor bottleneck (=2), significant bottleneck (=3), or very major bottleneck (=4). We first present the grading in heat maps according to the very major or significant health system bottlenecks as reported by all 12 countries, then by mortality contexts (neonatal mortality rate [NMR] <30 deaths per 1000 live births and NMR ≥30 deaths per 1000 live births) and then by region (countries in Africa and countries in Asia) (Figure 2a-c). We developed a second heat map showing the specific grading of bottlenecks for each health system building block by individual country (Figure 3a-c).

Finally, we categorised context specific solutions from the countries into thematic areas linked to the specific bottlenecks (Tables 1 and 2). We undertook a literature review to identify further case studies and evidence-based solutions for each defined thematic area (Additional file 2). For more detailed analysis of the steps taken to analyse the intervention specific bottlenecks, please refer to the overview paper [15]. The findings of the national MNH bottleneck analyses were also compared with results of the biennial WHO Maternal, Newborn, Child, Adolescent Health (MNCAH) policy surveys where information is collected from national Ministries of Health [16].

**Table 1:** Summary of solution themes and proposed actions for quality care during labour and birth (part A).

Health system building blocks	Solution Themes	Proposed actions from programme experience and literature review
	*Advocacy and political will*	• Active involvement and coordination from national advocates (academic and professional bodies, policy makers, hospital management committees) on quality care for labour & birth and emergency obstetric care.
Leadership and Governance	*Review and disseminate policies and guidelines*	• Develop a unified national implementation plan for SBA, BEmOC and CEmOC.
		• Improve context specific planning and policy on referral systems for births, birth companionship and standard operating guidelines for different level facilities, including the private sector.

	*Budget allocation*	• Prioritise, increase and sustain funding for emergency obstetric care to ensure multi-year predictable financing of services based on need.
Health Financing	*Innovative funding and removal of user fees*	• Ensure there is accountability and in-built mechanisms to minimise financial corruption at the facility, local and national level.
		• Apply learning from existing schemes to reduce financial barriers to care-seeking, such as incentive and voucher schemes and consider public private partnerships.
		• Ensure existing systems cover care at birth including transport, referral and care for complications (e.g. caesarean section).

	*Human resource management*	• Develop clear job descriptions with appropriate remuneration mechanisms and career development pathways (e.g. national accreditation system for SBAs and a midwifery cadre).
	*Competency based training*	• Increase the number of sanctioned posts, including specialists, within the public sector and ensure systems exist for adequate recruitment, rational deployment and ongoing retention working towards universal skilled attendance.
Health Workforce		• Scale up of simplified, skills and competency based training programmes on basic emergency obstetric care, including assisted vaginal delivery and respectful care practices.
		• Where appropriate, involve the private sector in training programmes.
	*Task shifting*	• Maximise existing resources and assess competencies for lower level health workers to take on tasks such as assisted vaginal deliveries and anaesthesia.
	*Mentoring and supervision*	• Improve mentoring through robust performance monitoring and supervision systems for SBAs.

**Table 2:** Summary of solution themes and proposed actions for quality care during labour and birth (part B).

	*Essential medical list*	• Include drugs and commodities needed during labour and childbirth in the national supply lists for e.g.: partograph, vacuum extractor, oxytocin.
	*Logistics management*	• Strengthen logistics management systems and national capacity through use of appropriate and available communication technologies.
Essential Medical Products and Technologies	*Infrastructure and equipment*	• Institute centralised blood data storage and blood donation camps.
		• Ensure essential equipment is available for BEmoC at first level facilities including vacuum extractors and forceps.
		• Rationally expand number of caesarean section services across the country and provide caesarean section kits.
	*Increase service delivery*	• Expand the number of 24/7 services, especially the availability of BEmOC and assisted vaginal delivery services.
	*Quality of Care*	• Improve quality of care through improved mentorship and robust performance monitoring and supportive supervision systems for SBAs.
Health Service Delivery		• Improve remuneration and incentives (working hours, food provision) to improve working conditions, motivation and promote respectful care practices.
	*Strengthen referral care*	• Improve referral links and transportation systems through context based planning to ensure inequities in access are minimised.

	*Strengthen and integrate health management information systems*	• Strengthen vital registration systems at national and local level.
		• Improve reporting systems and tools to ensure data quality and build national capacity for data-driven decision making (e.g. dashboard).
Health Information System		• Institutionalise regular spot checks to see whether indications for caesarean section were followed.
		• Incorporate community and private facility data into national HMIS.
	*Perinatal death audits and registers*	• Institutionalise maternal and perinatal death audits and quality assurance mechanisms with full audit cycle based on action and accountability.

	*Health promotion, education, community engagement*	• Sensitisation and health education to improve demand for quality obstetric care, respectful care and access to skilled birth attendance and emergency obstetric care.
Community Ownership and Participation		• Develop innovative community partnership models and promote transparency and social accountability for obstetric services.
	*Male involvement*	• Promote male involvement through use of male role models, inclusive policies and more targeted health education.
	*Improve Referral linkages*	• Strengthen continuum of care from household to health facilities through functional communication, transport and referral services.
		• Establish functional communication, transport and referral services.

## Results

Our analysis identified bottlenecks across seven health system building blocks for essential care during childbirth and the immediate postnatal period. Workshop participants in 12 countries submitted their responses to the bottleneck survey tools for SBA, BEmOC and CEmOC. Afghanistan, Cameroon, Democratic Republic of Congo (DRC), Kenya, Malawi, Nigeria, Uganda, Bangladesh, Nepal and Vietnam returned national level responses. Pakistan provided subnational data from all provinces: Gilgit-Baltisan, Azad Jammu and Kashmir, Khyber Pakhtoonkhwa, Baluchistan, and Punjab except Sindh and consultations were not held in the tribal areas. Participants from India provided subnational data from three states: Andhra Pradesh, Odisha and Rajasthan. Afghanistan listed their bottlenecks and rated all building blocks, but did not propose any solutions. The detailed bottlenecks and solution themes across all building blocks are summarised in the Additional file 2.

Provision of quality care during childbirth is still a major challenge across most countries included in the assessment. Grading according to major and significant bottlenecks for SBA, BEmOC and CEmOC as reported by 12 countries is shown in Figure 2(a-c). Grading according to number of countries that reported very major or significant health system bottlenecks for SBA, BEmOC and CEmOC are shown in Figure 3(a-c) respectively. Overall, health system building blocks with most, very major or significant bottlenecks were health financing and health workforce for SBA; health financing for BEmOC and health financing and service delivery for CEmOC. As anticipated and similar to other intervention packages, countries with higher NMR generally rated building blocks as having significant or very major bottlenecks. African countries reported a greater number of significant or very major bottlenecks for all the 3 intervention packages - with Nigeria having the highest number of very major bottlenecks.

The section below discusses all health system bottlenecks and solutions for SBA, BEmOC and CEmOC.

## Leadership and governance bottlenecks and solutions

The first building block, leadership and governance, was considered a major bottleneck in six countries for SBA and BEmOC and in five countries for CEmOC. Participants from both Asian and African countries identified limited advocacy efforts, lack of effective leadership and political will as bottlenecks for scaling up quality essential services during labour and childbirth. Limited availability of evidence-based guidelines for EmOC, especially for private sector facilities was identified as a bottleneck. Participants also highlighted that national planning efforts are often not tailored to the local context. Other bottlenecks reported included the lack of supportive policies for birth companionship, task shifting for anaesthesia services and allowing nurses and midwives to carry out assisted vaginal deliveries (see Additional file 2).

Country teams identified numerous solutions to address leadership and governance bottlenecks for scaling up essential services during childbirth. For SBA, participants suggested that national authorities should be proactive to implement (develop, train, disseminate) evidence-based standards for essential care at all levels, including in the private sector. Teams suggested improved context-specific planning at all levels in order to reduce unmet need and address inequities by focusing on marginalised and hard-to-reach population groups. The need for rational deployment of SBAs, improved supervision and a re-organisation of BEmOC and CEmOC services based on geography, client volume and unmet need were identified by Nigeria and Pakistan as important solutions. In addition, country teams also suggested that a unified national plan to scale up EmOC services with robust monitoring of performance was required. High-level advocacy and coordination focused on improving quality of care at facilities is needed. For CEmOC, teams suggested development of standard operating guidelines, job aids, algorithms for caesarean sections and blood transfusions. Participants from India identified the need to design innovative policies for birth companionship at the state level, whereas, participants from Kenya identified permissive task shifting policies for anaesthesia and assisted vaginal deliveries as solutions to governance bottlenecks.

## Health financing bottlenecks and solutions

Health financing was identified as a major bottleneck by 10 countries for skilled care at birth, 8 countries for BEmOC and 9 countries for CEmOC services. Overall, participants from African countries graded health financing as a major bottleneck with all countries reporting this as a significant bottleneck for both skilled care at birth and BEmOC services. For CEmOC, 5 of the 6 African countries and 4 of the 6 Asian countries perceived that health financing was a significant bottleneck. Overall, participants perceived that national financing for essential childbirth services was inadequate, financial barriers to care seeking were widespread and the absence of adequate, multi-year, predictable financing limited the scale up of EmOC services. India, the most populous among these countries did not view health financing as a very major bottleneck across all intervention areas unlike Nigeria perhaps due to the increased political and financial investment to improve women's and children's health in recent years.

Solutions proposed included removal of financial barriers to care seeking by developing innovative financing mechanisms. These included universal health coverage (DRC), social protection schemes (Kenya), results-based financing mechanisms (Cameroon) and pay-for-performance models (Bangladesh). Teams suggested increased allocation of resources for EmOC so that essential care services including blood-banking facilities can be expanded nationwide. Strengthening of hospital management committees and mobilising additional resources locally were also identified as important solutions. Finally, participants also felt that national plans and strategies must have in-built transparency and accountability mechanisms so that misuse of resources and corruption is minimised.

## Health workforce bottlenecks and solutions

Health workforce was identified as having the most critical bottlenecks for skilled care at birth across most countries. For both BEmOC and CEmOC services, 8 countries, perceived health workforce as having significant bottlenecks. For the three intervention packages participants in all countries highlighted the uneven distribution of skilled health workers as a major bottleneck. Nepal and Pakistan reported significant bottlenecks due to inadequate production, ineffective deployment and poor retention of health workers especially in rural and remote areas. Malawi reported that up-to-date training guidelines and development of relevant job descriptions for various cadres of health workers was a challenge. Overall, countries reported lack of adequate production of trained human resources, lack of comprehensive national human resource plans, limited opportunities for further training and career progression, low salaries, poor work environments and limited supervision as critical bottlenecks. Poor quality of trainings and post training follow-up and supervision were also identified as a major bottleneck by country teams.

Solutions identified by country teams included developing appropriate job descriptions for health workers, designing appropriate career development pathways including adequate remuneration packages to attract and retain skilled providers. Participants also stressed upon the development of comprehensive national human resource plans with strategies to ensure adequate recruitment, rational deployment, ongoing retention and capacity building of SBAs. Increasing the number of sanctioned posts in the public sector was also identified as an important solution. Development of accreditation mechanisms for SBAs and design of appropriate, evidence-based EmOC trainings were also identified as important solutions. Scaling up production of SBAs including the creation of a midwifery cadre was identified as an important solution to ensure universal coverage. Promotion of woman centred care during labour and childbirth was reported as an important strategy to increase service utilisation and improve health worker satisfaction. Improvement of overall work climate at health facilities and design of innovative mechanisms to improve staff motivation through appropriate incentives was also identified as an important solution.

## Essential medical products and technologies bottlenecks and solutions

This building block was found to have less significant bottlenecks for skilled care at birth compared to other building blocks. For BEmOC and CEmOC, all African countries compared to just two Asian countries reported this as a significant bottleneck. Specifically, Malawi and Nigeria reported that BEmOC equipment and supplies are not provided through national supply mechanisms. Issues related to logistics and commodity management and maintenance were perceived as critical bottlenecks in Nigeria and Pakistan. In many of these countries, lack of nationally agreed minimum standards for drugs, supplies and equipment results in procurement from a variety of sources often leading to receipt of supplies of variable quality. Further, in most LMICs as national procurement and logistics management systems are weak, problems such as overstocking at the centre and stock-outs in districts occur. Country teams also reported that weak infrastructure was a major bottleneck for the provision of 24/7 EmOC signal functions.

Solutions identified included strengthening existing national logistics management systems to ensure adequate forecasting, supply and availability of essential drugs and commodities through the development of robust, web-based logistics management information systems. Country teams also identified that essential drugs, equipment and commodities specifically partograph, forceps and vacuum extractors must be included in national supply chains including at first level facilities. Establishment of centralised blood storage facilities and organisation of regular blood donation camps were identified as important solutions to improve availability of blood transfusion services. Expansion of CEmOC services including improving availability of specialists, provision of caesarean section kits and blood transfusion services free of cost were identified as an important solution.

## Health service delivery bottlenecks and solutions

The majority of countries (9 out of 12) identified health service delivery as a critical bottleneck for provision of both BEmOC and CEmOC services. A greater proportion of African countries (5 of 6) identified health service delivery as an important bottleneck for scaling up both BEmOC and CEmOC services. Overall country teams felt that there was a need to improve quality of care (QoC) during labour and childbirth including better intrapartum monitoring and strengthening referral linkages. Weak facility infrastructure was also identified as a major bottleneck for the provision of EmOC signal functions. Participants from Pakistan also highlighted that maternity services are not often user friendly and that provision of respectful maternity care is a major challenge.

Solutions identified by participants included improvement of QoC for facility based obstetric and neonatal care services, improved intrapartum monitoring and institutionalisation of quality improvement mechanisms such as maternal and perinatal death audits. Participants highlighted the need to expand the availability of 24/7 EmOC services, specifically assisted vaginal deliveries and caesarean sections and improve referral and transportation services. Improved context-specific planning and capacity building of district health managers to conduct evidence based planning was also found to be an important solution. Participants also felt that public-private partnership mechanisms should be explored to expand availability and access to EmOC services.

## Health information systems bottlenecks and solutions

Health information systems were not considered to have as many major or significant bottlenecks for the three interventions across countries or regions. Overall, countries felt that national Health Management Information Systems (HMIS) needed improvement and that existing systems lacked standard indicators and harmonised recording tools for monitoring EmOC programme performance.

Strengthening of routine monitoring of maternal and newborn health programmes with an emphasis on improving data quality and building capacity for data driven decision making at all levels of the health system was identified as an important solution. The need to incorporate data from community-based programmes and private sector facilities into national HMIS system was also identified as an important solution. Participants proposed the development of a maternity dashboard system with standard indicators to monitor EmOC performance at the district level and establishing a national maternal and perinatal death surveillance system. Given increasing Caesarean section rates across all countries, participants also identified the need to establish a quality assurance and monitoring system to verify whether indications for caesarean sections are being followed.

## Community ownership and partnership bottlenecks and solutions

Community ownership and partnership was not considered to have as many major or significant bottlenecks for the three interventions across countries or regions. Overall, country teams perceived that health promotion, education and community engagement in RMNH programmes needed further improvement. Lack of coordination between front line health workers and community structures seems to be a major bottleneck in Nigeria. Challenges with design of suitable communication materials, lack of male involvement and poorly designed information, education and communication tools was reported by most of the countries. Poor referral linkages between first level and tertiary level facilities were also identified as a critical bottleneck.

Solutions identified included increasing the emphasis on health education, promotion, and demand-creation activities to improve care seeking for quality services during labour and childbirth. Participants also emphasised the need to design innovative community partnership models that strengthen accountability and transparency of efforts. Improving the continuity of care from household to health facilities, promotion of male involvement and establishing functional communication, transport and referral services were also identified as important solutions for improving essential care during labour and childbirth.

## Discussion

This paper presents an analysis and synthesis of bottlenecks and solutions for three intervention areas (SBA, BEmOC, CEmOC) related to the provision of quality essential care during labour and childbirth. According to previous analysis from the Lancet *Every Newborn *series, Lancet Midwifery series and the *Every Newborn *action plan, although care at the time of birth has the greatest potential to improve maternal and newborn survival, there is low and inequitable coverage including a quality gap in the provision of care at the time of birth [14,17]. Our analysis shows that provision of quality care during labour and childbirth is intrinsically related to the functionality of the overall health system, specifically health financing, human resources and health service delivery building blocks. The availability of effective intervention packages and high level political commitment to the reduction of maternal and newborn deaths has shown good results so far. However, we have found that long-term investment and planning to strengthen health systems especially human resources has been lacking from national and global responses.

The methodology used for analysis is unique as data were captured through a bottom-up, consultative process to capture the context specific nuances related to field implementation. The bottlenecks identified by country teams are the bottlenecks to implementation as perceived by frontline workers and agencies delivering services on the ground. The MNH bottleneck analysis tool was comprehensive and facilitated the identification of relevant bottlenecks associated with essential care during labour and childbirth. In addition, an innovative structure of the tool meant that consensus had to be generated amongst participants to obtain a rating for each health system bottleneck. Our analysis identified three priority health system building blocks for intrapartum care: health financing and health workforce for skilled care at birth, health service delivery for BEmOC, health financing and service delivery for CEmOC. The solution themes including priority actions identified from literature review and programme learning are shared below.

### Health-financing priority actions

The provision of high quality maternity services requires adequate financing for operations, staff, medicines, supplies, equipment and food. Seeking care at facilities also has implications for households in terms of transport costs, patient and their companions' time and their time away from work. Childbirth is the most expensive period during the entire pregnancy and in case of complications, households may even have to bear catastrophic expenses [15,18]. The Global Investment Framework for Women's and Children's Health states that an increase in health expenditure for maternal and newborn health at the country level of US$ 5 per capita per year until 2035 could result in a nine- fold returns on overall economic and social benefits [19]. Investment gaps for health systems strengthening in countries are well known [19]. The package for essential care during labour and childbirth is cost-effective and needs to be supported by public financing. Two priority areas seem to be crucial for health financing; the removal of financial barriers to care seeking and strengthening national financing.

#### Removal of financial barriers to care seeking

Financial barriers to care seeking during pregnancy and childbirth are still widespread with high out of pocket expenses for childbirth services [20,21]. The ability to pay is a significant determinant for utilisation of institutional deliveries. A recent study in India showed that the mean out-of-pocket expenditure on a normal delivery in a public facility was US$28 compared with US$84 in a private facility, and caesarean delivery costs three times more than a normal delivery [22]. Many countries in Asia and Africa have pursued user fee removal or fee exemption for care during labour and childbirth including for caesarean section [23]. Most studies reviewing utilisation following the removal of user fees for deliveries have found a rise in assisted deliveries and caesarean sections at health facilities [21,24-26] and, in some cases, found that gains are greater in poorer groups [27].

Many promising strategies such as the development and expansion of community-based insurance schemes, voucher schemes, conditional cash transfers and demand side financing exist. Available evidence also indicates the need for careful planning of the supply side in order to cope with increased demand after removal of user fees or demand side barriers [26,28-32]. Regardless of the strategy adopted to remove barriers to access and utilisation of care, clear guidelines for effective implementation, long term sustainability, and efficient and transparent management procedures must be developed [33-35]. Campaigns to increase public awareness about these schemes and innovative strategies targeting hard to reach, poor and marginalised groups will help to increase utilisation rates [14].

#### Strengthen national financing

Many countries reported that national budgets allocated to maternity care including essential care and care for complications are inadequate, high out of pocket expenses are widespread and lack of innovative financing models are important barriers for scaling up of EmOC services. Ensuring sufficient funding requires strengthening of national financing systems through innovative mechanisms that provide greater value for money, increase resource mobilisation and effective mechanisms to ensure accountability of resources and results. Specifically, increasing investments through development of innovative financing models, subsidies and local fund generation mechanisms (pool funding, cost sharing or revolving funds) could be potential solutions. Evidence exists to show that MNCH outcomes can be improved by making service delivery more effective, efficient and equitable [36]. Efficiency gains can be made through adoption of promising strategies such as sector wide approaches to improve harmonisation and coordination of efforts in countries. Innovative financing mechanisms such as performance based financing mechanism are also promising approaches [37]. Decentralisation may also be a more efficient way for utilising existing resources. Prepayment schemes such as social health insurance are also effective in improving service utilisation and reducing catastrophic health expenses [38]. Demand-side financing mechanisms such as conditional cash transfers and vouchers have also shown promising results in Latin America and India [39,40]. Equity gains may be achieved through subsidies for drugs and commodities that reduce the cost price of these commodities. National governments should increase funding for essential care during labour and childbirth by developing appropriate investment plans, leverage additional funds from existing RMNCH programmes, design, implement and expand pro-poor strategies and legislation to improve access and reduce catastrophic health expenses.

### Health workforce priority actions

Health workforce is an integral component of any functional health system and essential for providing quality health services. A worldwide shortage of skilled birth attendants including midwives exists as a result of multiple and complex factors [41]. We know that MNCH outcomes tend to be better in countries with greater numbers of skilled health workers [41]. Availability and distribution of SBAs varies considerably amongst and within countries. Highly skilled personnel, such as obstetricians, paediatricians, anaesthetists, midwives and others tend to be concentrated in urban areas, capitals or tend to migrate overseas to pursue better prospects. This is especially relevant in African countries. For example, DRC reported lack of skilled personnel in rural areas as a major bottleneck. Recent estimates indicate that in 53 of the 68 priority countdown countries, the density of doctors, nurses and midwives is below the WHO recommended threshold [42,43]. Human resource challenges in LMICs include inadequate production, ineffective deployment, and poor retention, lack of supervision, poor salaries, low morale and motivation and lack of infrastructure for SBAs [44-46].

Many countries are now rapidly scaling up production of midwives [47] as evidence exists to show that midwife-led continuity of care models help to normalise childbirth [48], are equally effective as other models of medical-led care and shared care [48] and lead to better maternal, perinatal and neonatal outcomes including prevention of stillbirths and other maternal and neonatal complications [17,48]. Low and middle-income countries such as Burkina Faso, Cambodia, Indonesia, and Morocco have demonstrated reduction of maternal and newborn mortality through deployment of midwives [49]. Educated, licenced and well supported midwives, including nurse midwives trained to international standards in midwifery possessing the required competencies are needed across all settings [17]. We suggest that countries need to develop long term human resource plans (5 to 10 year) that outline detailed strategies for training, distribution and retention of health workers particularly midwives, neonatal nurses and neonatologists.

**Figure 4:**
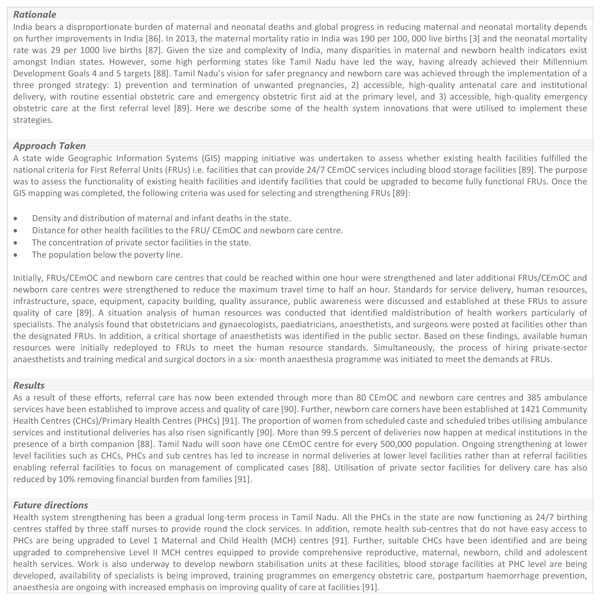
**Health system strengthening for improving maternal and newborn health service delivery in Tamil Nadu, India**. GIS: geographical information systems. FRUs: first referral units. CEmOC: comprehensive emergency obstetric care. CHCs: community health centres. PHCs: primary health centres. MCH: maternal and child health.

Effective human resource management requires strong leadership and comprehensive human resource planning guided by actual needs on the ground. Countries should undertake or update national human resource analyses to map existing SBAs, especially midwives, both in the public and private sectors, identify gaps in EmOC service provision, any ongoing task-shifting or quality improvement mechanisms. Figure 4 provides a case study of such an approach employed in Tamil Nadu. Appropriate strategies to ensure training quality, rational deployment, retention, skill mix and appropriate regulation of health workers is also needed. Innovative incentive mechanisms such as performance-based payments or hardship allowances for rural postings can also be introduced. Two areas of importance for this building block are training quality and task shifting.

#### Training quality

Countries perceived that poor quality of training is a major bottleneck and identified that evidence based training techniques (for e.g.: competency based trainings) rather than didactic, lecture-style trainings focused on skill acquisition are needed. Malawi reported that up-to-date training guidelines and development of relevant job descriptions for various cadres of health workers was a challenge (see Additional file 2). Overall, countries need to adapt and implement globally recommended, evidence-based clinical guidelines for EmOC, strengthen both pre-service and in-service training, improve post-training mentorship, and assure quality of services. A systematic approach to harmonised, standardised and competency-based training that is needs-driven and accredited is essential. Training and retraining programmes should be linked with certification, registration and career progression mechanisms that are standardised and nationally endorsed. More evidence is needed to inform effective ways of scaling up the midwifery workforce particularly in terms of the education, regulation, in-service training, career progression, deployment, and retention and increasing of the quality, relevance, and productivity of midwives across public, private, and not-for-profit sectors [47].

#### Task shifting

Given shortages of skilled health workers and skill-mix disparities between available health workers, task shifting has emerged as an important strategy to delegate tasks to lower level cadres by providing them focused training on key activities [50]. Task shifting has been implemented successfully to improve access, better utilise existing resources and implement programmes for improving maternal and child health by using community health workers for diverse programmes such as immunisation, family planning, obstetric surgery and management of childhood illnesses [51,52]. Non-physician clinicians have also been utilised in many African countries to provide EmOC services including caesarean sections [53-59]. Detailed recommendations on optimising health worker roles to improve access to key MNH interventions through task shifting are available [60]. Countries facing severe shortages of SBAs should consider implementing a task shifting approach to EmOC services and develop appropriate legislative and regulatory frameworks. Supportive supervision and ongoing clinical mentoring is essential whenever task shifting is employed and should be done by competent health workers that have the appropriate supervisory skills.

### Health service delivery priority actions

Health service delivery was found to be a major bottleneck for BEmOC and CEmOC services, and although not included in the critical 'red' category for skilled care at birth; 8 of the 12 countries identified significant and very major bottlenecks. As the tracer indicator for BEmOC services was the use of assisted vaginal deliveries, which depends on national legislative, regulatory environment as well as availability of vacuum or forceps, this may have falsely denoted problems with BEmOC service delivery. Assisted vaginal deliveries are not available in most of the African settings. A recent study in Zambia found that only 12% of facilities in the country could be classified as being at least BEmOC-1 (with the exception of assisted vaginal deliveries) and that majority of facilities in the country did not provide the recommended BEmOC signal functions [61]. Results from the WHO global surveys also found that overall rates for assisted vaginal delivery were low at 3·2% in Asia [62] and 3% in Africa [63]. Compared to caesarean sections, vacuum extraction can be performed by nurses and midwives and they are safer for future pregnancies, hence additional efforts are needed to promote these assisted methods. For BEmOC services, some Asian countries reported that midwives, nurses and Lady Health Visitors are not authorised to perform assisted vaginal deliveries or prescribe oxytocin. Specifically, Vietnam and Pakistan reported that standard guidelines for the use of partograph and use of assisted vaginal deliveries were not available (see Additional file 2). Asian countries tend to perceive that lack of specific standards for EmOC is a bottleneck whereas African countries perceive that dissemination of these normative documents is the more significant bottleneck. This is in contrast to the findings of the WHO MNCAH policy surveys where all countries except Nigeria reported availability of national guidelines for pregnancy, childbirth, postpartum and newborn care [16]. This discrepancy could perhaps be explained by the fact that different data collection methods were utilised; the MNCAH policy surveys rely on national authorities to report to the WHO, whereas the every newborn consultation utilised a more qualitative approach. Lack of up-to-date clinical protocols and regulatory and legislative restrictions meant that assisted vaginal deliveries are omitted from training manuals such as in Malawi. Two important themes emerged as priorities for improving service delivery.

#### Quality of care

The quality of care (QoC) offered at maternity facilities affects pregnant women, both physically and emotionally, but also impacts the survival and long-term health of the mother and her newborn [64-66]. Ensuring high QoC is often complex [67], as conceptually, QoC comprises provision of timely, reliable, equitable, efficient, compassionate, patient-centred care, with application of evidence-based standards to ensure patient safety and health worker satisfaction [68-72]. Providing woman-centred care is central to ensuring high QoC at health facilities. Fear of disrespect and abuse by the health care provider is a powerful deterrent to the use of skilled care during childbirth [73]; and women's experiences with maternity services can have lasting impact on future utilisation of services. Given these challenges, experts have argued that QoC has remained a neglected agenda [74] and advocated for a renewed emphasis on quality improvement especially for care at birth [75]. Further, measurement of QoC is a major challenge across countries and efforts are underway to develop appropriate metrics [76].

**Figure 5:**
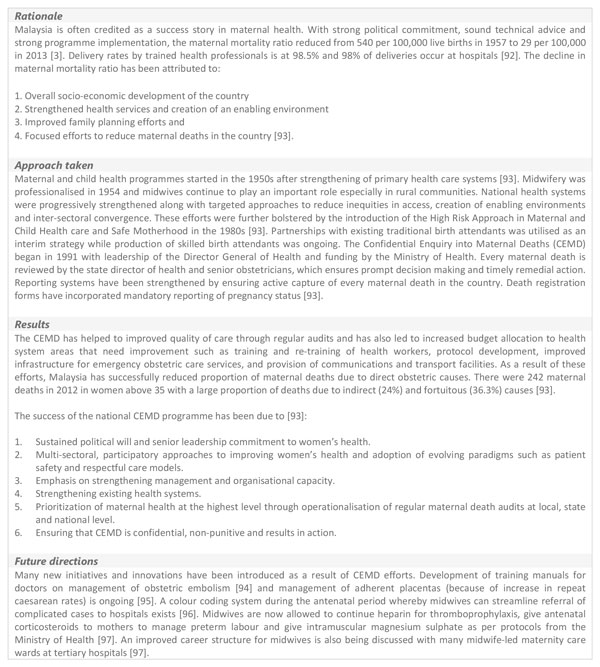
**Malaysia's approach to improving the quality of maternal and newborn health**. CEMD: Confidential Enquiry into Maternal Deaths

Many methods have proved successful in improving QoC for MNH. These include mortality audits or reviews for maternal and perinatal deaths (stillbirths and newborn deaths), review of cases of 'near-miss' or severe acute maternal morbidity (SAMM) and standards-based (or clinical) audit [77]. Figure 5 outlines a case study of Malaysia's approach to improving quality of care through a Confidential Enquiry into Maternal Deaths (CEMD) programme in Malaysia.

Although, all countries face different challenges to ensure QoC, the best approach seems to be through multifaceted interventions tailored to individual contexts supported by a progressive policy environment where quality assurance is central (Figure 4 and 5). Furthermore, health system strengthening through upgrading of infrastructure and services, an enhanced focus on monitoring, feedback and supervision, continued professional trainings and involving communities to demand for quality services is likely to result to overall improvements in QoC.

#### Public private partnership

Rapid population growth, increasing urbanisation, increase in inequities has meant that many public health systems have not been able to cope with increasing demands placed on health systems. Public private partnership models could be a potential strategy to tackle these challenges. In many African countries, faith based organisations contracted by the ministry of health provide a significant proportion of labour and childbirth services [78]. Although, many critics have urged caution as private sector facilities may overprescribe diagnostics, procedures and medicines [79,80], they have emerged as an important provider of delivery services in many LMICs [81]. An innovative scheme in Gujarat, India known as the "Chiranjeevi Yojana", involved contracting out to private sector obstetricians to provide delivery care services and targeted poor women in rural areas. Results showed that clients saved around US $82 for delivery compared to those that did not benefit from the scheme [82]. It has also led to an increase in institutional delivery rates from 27% to 53% [83]. However, there are many evidence gaps regarding private sectors role in provision of EmOC services. Additional research is needed in countries to understand the role of the private sector in providing EmOC services, geographic distribution of facilities, types of services offered and their quality and legal and regulatory frameworks required.

### Other priority actions

As highlighted in the analysis and in tables 1 and 2, very major or significant bottlenecks were reported across all building blocks. Solution themes for three critical ones have been discussed above. However, a few other bottlenecks were identified for ensuring quality essential care during childbirth and solutions proposed for them. Leadership and governance solutions included high level advocacy and political commitment, design of progressive policies on birth companionship and task shifting, improved planning, supervision and mentoring of SBAs. Evidence exists to show that companionship during labour and continuous support of pregnant women improves chances of spontaneous vaginal births and also leads to improved satisfaction of women with maternity services [84]. Essential medical products and technology solutions included improving overall national logistics management capacity and strengthening facility infrastructure. Health information system solutions included strengthening of the HMIS, building national capacity for data driven decision making and scaling up quality improvement mechanism such as maternal and perinatal death audits, discussed in more detail elsewhere in the series [85].

### Limitations

The data generated from the workshop was based upon reaching a consensus amongst the participants. Hence, the quality and amount of information extracted from these workshops varied depending on the level of knowledge of participants on health system issues and facilitation skills. In addition, bottlenecks were reported as perceived bottlenecks relative to the other health system building blocks under exploration. There may be instances where known health system challenges or deficits based on robust quantitative data may be in conflict with the perceived bottleneck grading. This may be due to the method of grading relative to other health system building blocks, or that participants place higher subjective value on other areas of their health system. An additional explanation is that groups may view certain building block areas as easier challenges to overcome based on their knowledge of their setting and expertise in the specific technical intervention being discussed. The tool is comprehensive and detailed - which is one of its strengths. However, it also may have caused some *workshop fatigue*, particularly towards the end of the workshop. For example, Afghanistan completed the bottleneck portion of the questionnaires, but did not submit any solutions. Our analysis focused on three intervention packages necessary for quality essential care during childbirth and hence the discussion may have lost some of the nuances associated with implementation of individual interventions.

### Future agenda

Achieving high coverage of recommended maternal and newborn health interventions is important, but ensuring availability of good quality essential and emergency obstetric and neonatal care services is crucial for further improvements in maternal and neonatal outcomes. Although challenges vary amongst countries, a multi-faceted, health-systems approach supported by an enabling policy environment seems to be the most promising (Figure 4 and 5). Investments across health system building blocks on the pathway towards progressive universal health coverage in countries are necessary. Specific areas for action have been highlighted in the paper above. However, a renewed emphasis on quality improvement especially during labour and childbirth is needed with linkages to the Every Mother and Every Newborn package [15].

## Conclusions

**Figure 6:**
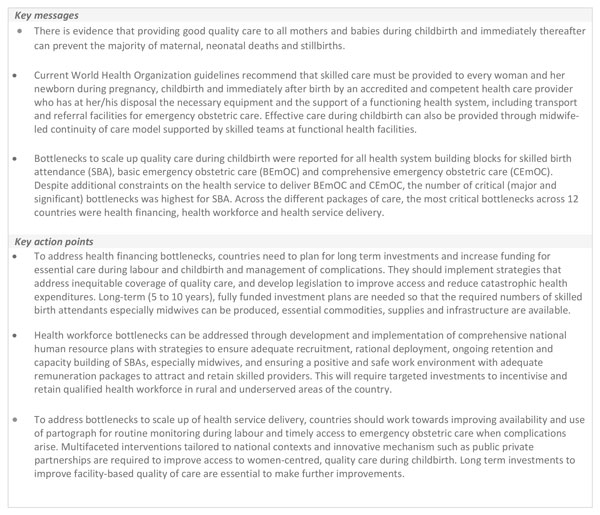
**Key messages and key action points for quality essential care during labour and birth**. SBA: skilled birth attendant. BEmOC: basic emergency obstetric care. CEmOC: comprehensive emergency obstetric care.

Whilst major bottlenecks to scaling up essential care for mothers and babies during childbirth still exist, there are many effective solutions to overcome these challenges. Figure 6 outlines the key messages and the key action points to improve quality care during labour and childbirth. A multi-dimensional approach to end preventable maternal and neonatal deaths, and stillbirths focused on design of context-specific solutions with tailored implementation seems promising. Our findings show that longer term investments (5 to 10 year plans) in health workforce, especially midwifery, and strengthening of essential obstetric and neonatal care services particularly through focussed investments for improving quality of care at time of birth are needed. Further progress will depend upon addressing inequities in access, utilisation and improving quality of care at facilities. Strengthening health systems to respond to the needs of mothers and newborns along a pathway of progressive universal health care in countries with a strong focus on accountability to improve quality of care and equity will be essential to end preventable maternal and neonatal deaths, and stillbirths.

## List of abbreviations

BEmOC: Basic Emergency Obstetric Care; CEmOC Comprehensive Emergency Obstetric Care; DRC: Democratic Republic of Congo; EmOC: Emergency Obstetric Care; HMIS: Health Management Information Systems; LMICs: Low and Middle Income Countries; MMR: maternal mortality ratio; MNCAH: Maternal, Neonatal, Child and Adolescent Health; NGOs: Non-governmental organizations; NMR: neonatal mortality rate, SBA: skilled birth attendants; QoC: Quality of Care; WHO: World Health Organization.

## Competing interests

The authors have not declared competing interests. The assessment of bottlenecks expressed during consultations reflects the perception of the technical experts and may not be national policy. The authors alone are responsible for the views expressed in this article and they do not necessarily represent the decisions, policy or views of the organisations listed, including WHO.

## Authors' contributions

UNICEF and ENAP teams were responsible for the overall coordination of the country consultation process, data compilation and bottleneck analysis tool development. GS was responsible for the analysis and overall writing process. MM oversaw the analysis, writing and reviewed numerous versions of the draft paper. AW, KED, JH, JEM, LTD, SF, TL, ASK, LdB contributed sections of text and reviewed numerous drafts. All named authors contributed to the final draft of the paper and approved the final manuscript.

## Supplementary Material

Additional file 1 Bottleneck tool questionnaire. Click here for file

Additional file 2 Supplementary tables, figures and literature search strategy. Click here for file

## Declarations

Publication costs for this supplement were funded by the Bill and Melinda Gates Foundation through a grant to US Fund for UNICEF (Grant ID: OPP1094117), and support from Save the Children's Saving Newborn Lives Programme. Additional funding for the bottleneck analysis was received from USAID (Grant ID: GHA-G-00-07-00007) through UNICEF.

This article has been published as part of *BMC Pregnancy and Childbirth *Volume 15 Supplement 2, 2015: Every Woman, Every Newborn. The full contents of the supplement are available online at http://www.biomedcentral.com/bmcpregnancychildbirth/supplements/15/S2.
