# Supplementary material for: Quality care during labour and birth: a multi-country analysis of health system bottlenecks and potential solutions
Source: BMC Pregnancy Childbirth. 2015 Sep 11;15(Suppl 2):S2. doi: 10.1186/1471-2393-15-S2-S2 (PMC4577867; doi:10.1186/1471-2393-15-S2-S2)
Supplement: Additional file 2 — Supplementary tables, figures and literature search strategy. [file 1471-2393-15-S2-S2-S2.docx]

Quality care during labour and birth: a multi-country analysis of health system bottlenecks and potential solutions

Additional file 2

[A. Table S1: Bottlenecks for quality care during labour and birth for SBA 2](#_Toc421175851)

[B. Table S2: Bottlenecks for quality care during labour and birth for BEmOC 7](#_Toc421175852)

[C. Table S3: Bottlenecks for quality care during labour and birth for CEmOC 10](#_Toc421175853)

[D. Table S4: Bottlenecks for quality care during labour and birth for 2 or more interventions 13](#_Toc421175854)

[E. Table S5: Solutions for quality care during labour and birth for SBA 26](#_Toc421175855)

[F. Table S6: Solutions for quality care during labour and birth for BEmOC 40](#_Toc421175856)

[G. Table S7: Solutions for quality care during labour and birth for CEmOC 54](#_Toc421175857)

[H. Table S8: Bottleneck themes and solutions identified for SBA, BEmOC and CEmOC 70](#_Toc421175858)

[I. Figure S1: Subnational grading of bottlenecks for quality care during labour and birth for SBA, BEmOC and CEmOC 78](#_Toc421175859)

[J. Literature search strategy 80](#_Toc421175860)

[K. References 81](#_Toc421175861)

# A. Table S1: Bottlenecks for quality care during labour and birth for SBA

| Healthy System Building Block | Category | Bottlenecks | Africa | | | | | | Asia | | | | | | |
| --- | --- | --- | --- | --- | --- | --- | --- | --- | --- | --- | --- | --- | --- | --- | --- |
|  |  |  | Cameroon | DRC | Kenya | Malawi | Nigeria | Uganda | Afghanistan | Bangladesh | India | Nepal | Pakistan | Vietnam |  |
| Leadership and Governance | **Policy/strategy** | Policy not based on evidence/data |  |  |  |  |  |  |  |  |  |  | S |  |  |
|  |  | No professional midwives |  |  |  |  |  |  |  |  |  | ✓ |  |  |  |
|  | **Guidelines / standards**  **Protocols** | Traditional Birth Attendants and Private hospitals not under partograph guidelines |  |  |  |  |  |  |  |  | O |  |  |  |  |
|  | **Coordination / management** | Inadequate coordination on Maternal Newborn Health issues i.e. in-service trainings conducted by partners |  |  |  | ✓ |  |  |  |  |  |  |  |  |  |
|  | **Public-private partnership** | No adequate public private partnership |  |  |  |  | ✓ |  |  |  |  |  | A, B |  |  |
|  | **Accountability** | Lack of accountability and prioritisation | ✓ |  |  |  |  |  |  |  |  | ✓ | G, K |  |  |
|  | **Engagement** | Ineffective engagement of key stakeholders like civil society organization etc. |  |  |  | ✓ |  |  |  |  |  |  |  |  |  |
|  | **Funding** | High dependence on financial support from partners |  | ✓ |  |  |  |  |  |  |  |  |  |  |  |
|  |  | Inadequate coverage of output based aid/financing |  |  | ✓ |  |  | ✓ |  |  |  |  |  |  |  |
|  | **Financial barriers to care** | Lack of (results-based) financial mechanism |  |  |  |  |  |  |  |  |  |  | A, B, K, P, S | ✓ |  |
|  |  | High cost for poor quality |  |  |  |  |  |  |  |  |  | ✓ |  |  |  |
| Health Workforce | **Training** | Lack of systematic development of curriculums |  |  |  |  |  |  |  |  |  |  | S |  |  |
|  |  | Poor quality of training (materials and practices) and lack of monitoring |  |  |  |  |  |  |  |  |  | ✓ |  |  |  |
|  | **Human resources strategy** | No service delivery plan for utilization of skilled providers |  | ✓ |  |  |  |  |  |  |  |  |  |  |  |
|  | **Job descriptions/ aids** | Guidelines and job aids are not used or adhered to |  |  |  | ✓ |  |  |  |  |  |  |  |  |  |
| Essential Medical Products and Technologies | **Essential Medical List (EML)** | Lack of Essential Medicines List categorisation |  |  |  |  |  |  |  |  | A |  |  |  |  |
|  | **Procurement and supply management** | Existence of several informal sources for medical products and technologies |  | ✓ |  |  |  |  |  |  |  |  |  |  |  |
|  |  | In many areas, procurement supported by development partners |  |  |  |  |  | ✓ |  |  |  |  |  |  |  |
| Health Service Delivery | **Service availability / capacity of services** | System not user-friendly |  |  |  |  |  |  |  |  |  |  | K |  |  |
|  |  | Skilled birth not available in rural health zones |  | ✓ |  |  |  |  |  |  |  |  |  |  |  |
|  | **Management** | Poor health facility management |  |  |  |  |  |  |  |  |  | ✓ | A, P |  |  |
|  | **Referrals** | Regulation for referral is available, but not functioning |  |  |  |  |  |  |  |  |  |  |  | ✓ |  |
|  | **Quality of care/ quality improvement** | System not user-friendly |  |  |  |  |  |  |  |  |  |  | K |  |  |
|  | **Communication and health worker attitudes** | Provider reluctance to use partograph |  |  |  |  |  |  |  |  |  |  | G |  |  |
|  | **Policy** | Policy not based on evidence/data |  |  |  |  |  |  |  |  |  |  | S |  |  |
| Health Information System  Health Information System | **Tools for information system/ reporting** | Non-functional health card | ✓ |  |  |  |  |  |  |  |  |  |  |  |  |
|  |  | Difficult recovery of data | ✓ |  |  |  |  |  |  |  |  |  |  |  |  |
|  |  | Information system not responsive to particular needs in area |  |  |  |  |  |  |  |  |  |  | S |  |  |
|  | **Indicators** | Community based data not captured in the Health Management Information Systems |  |  |  |  | ✓ |  |  |  |  |  |  |  |  |
|  |  | No consensus on the concept of “skilled birth attendant” yet |  |  |  |  |  |  |  |  |  |  |  | ✓ |  |
|  | **Use and dissemination of information** | Data is currently being documented, but use for implementation, learning, planning and delivery of services is limited |  |  | ✓ | ✓ |  | ✓ |  |  |  |  |  |  |  |
| Community Ownership and Partnership  Community Ownership and Partnership | **Promotion / communication** | Lack of coordination |  |  |  |  | ✓ |  |  |  |  |  |  |  |  |
|  |  | Limited implementation of social audit |  |  |  |  |  |  |  |  |  | ✓ |  |  |  |
|  | **Care-seeking** | Inadequate health care-seeking behaviour because of insufficient programs and inadequate Community Health Workers resulting in poor Infection Prevention & Control/Behaviour Change Communication with mother /families |  |  |  |  |  |  |  | ✓ |  |  |  |  |  |
|  |  | Geographical difficulties preventing mothers’ use health services |  |  |  |  |  |  |  |  |  |  |  | ✓ |  |
|  | **Barriers / challenges faced by mothers** | Poor public perception and confidence in quality of services |  |  |  |  |  |  |  | ✓ |  |  |  |  |  |
|  |  | Language barriers |  |  |  |  |  |  |  |  |  |  |  | ✓ |  |
|  | **Community involvement and mobilization** | Limited willingness from health system to involve the community – transparency issues |  |  |  |  |  |  |  |  |  | ✓ |  |  |  |

# B. Table S2: Bottlenecks for quality care during labour and birth for BEmOC

| Healthy System Building Block | Category | Bottlenecks | Africa | | | | | | Asia | | | | | |
| --- | --- | --- | --- | --- | --- | --- | --- | --- | --- | --- | --- | --- | --- | --- |
|  |  |  | Cameroon | DRC | Kenya | Malawi | Nigeria | Uganda | Afghanistan | Bangladesh | India | Nepal | Pakistan | Vietnam |
| **Leadership and Governance** | **Policy/strategy** | Midwifes / Lady Health Visitors / Nurses not authorized for assisted vaginal deliveries and prescription/ administration of oxytocin during childbirth – only doctors |  |  |  |  |  |  |  |  |  | ✓ | B, S | ✓ |
|  |  | Lack of policy – only donor funded programs |  |  |  |  |  |  |  |  |  |  | B, G |  |
|  |  | Poor implementation of policy |  |  |  |  |  |  |  |  |  |  | S |  |
|  |  | Lack of leadership in implementation of strategies and policies |  |  | ✓ |  |  |  |  |  |  |  |  |  |
|  | **Guidelines/ standards/ protocols** | No extension of the normative documents available |  | ✓ |  |  |  |  |  |  |  |  |  |  |
|  |  | Lack of BEmOC protocols | ✓ |  |  |  |  |  |  |  |  |  |  |  |
|  |  | Clinical protocols have omitted assisted vaginal delivery |  |  |  | ✓ |  |  |  |  |  |  |  |  |
|  | **Awareness** | Inadequate awareness of policies by service providers (all cadres SBA) |  |  | ✓ |  |  |  |  |  |  |  |  |  |
|  | **Funding** | Lack of government subsidies for skilled delivery |  | ✓ |  |  |  |  |  |  |  |  |  |  |
| **Health Workforce** | **Training** | Most of the staff are trained in urban facilities |  | ✓ |  |  |  |  |  |  |  |  |  |  |
| **Essential Medical Products and Technologies** | **Essential Medical List (EML)** | No essential list on devices |  |  |  | ✓ | ✓ |  |  |  |  |  |  |  |
|  | **Procurement and supply management** | Inadequate maintenance |  |  |  |  |  |  |  |  |  |  |  |  |
|  |  | Centralised procurement system |  |  |  |  |  |  |  |  |  | ✓ |  |  |
|  |  | Weak logistics between state and facilities |  |  |  |  | ✓ |  |  |  |  |  |  |  |
|  |  | Informal sources of supply beyond official control |  | ✓ |  |  |  |  |  |  |  |  |  |  |
| **Health Service Delivery**  **Health Service Delivery** | **Service availability / capacity of services** | Coverage of community health services is inadequate and especially for maternal, newborn health |  |  | ✓ |  |  |  |  |  |  |  |  |  |
|  | **Referrals** | Weak community and facility linkage |  |  | ✓ | ✓ |  |  |  |  |  |  |  |  |
|  | **Quality of care/ quality improvement** | Standard treatment guidelines / protocols for BEmOC (e.g. no use partograph, no recording, poor essential newborn care, etc.) are not followed |  |  |  |  |  |  |  |  |  |  | B | ✓ |
|  | **Communication and health worker attitudes** | Poor staff attitude |  |  | ✓ |  |  |  |  |  |  |  | B |  |
|  |  | Provider reluctance to perform assisted vaginal delivery |  |  |  |  |  |  |  | ✓ |  |  |  |  |
|  | **Private sector** | High costs of services in private sector |  |  |  |  |  |  |  |  |  |  | B |  |
| **Community Ownership and Partnership** | **Barriers / challenges faced by mothers** | People consider Maternal Child Health is just a responsibility of health workers |  |  |  |  |  |  |  |  |  |  |  | ✓ |
|  | **Access** | Use of traditional birth attendants delaying access to care | ✓ |  |  |  |  |  |  |  |  | ✓ |  |  |

# C. Table S3: Bottlenecks for quality care during labour and birth for CEmOC

| Healthy System Building Block | Category | Bottlenecks | Africa | | | | | | Asia | | | | | |
| --- | --- | --- | --- | --- | --- | --- | --- | --- | --- | --- | --- | --- | --- | --- |
|  |  |  | Cameroon | DRC | Kenya | Malawi | Nigeria | Uganda | Afghanistan | Bangladesh | India | Nepal | Pakistan | Vietnam |
| Leadership and Governance | **Policy/strategy** | The rigid policy of using only blood supplied by blood bank for transfusion |  |  |  |  |  |  |  |  |  |  |  | ✓ |
|  |  | Specific actions for scaling up CEmOC are not explicit in the available strategies |  |  | ✓ |  |  |  |  |  |  |  |  |  |
|  |  | Centralized policies (eg non-recruitment) impact availability of services |  |  |  |  |  | ✓ |  |  |  |  |  |  |
|  | **Guidelines / standards/ protocols** | There are no SOPs for most surgical procedures |  |  |  | ✓ |  |  |  |  |  |  |  |  |
|  | **Coordination/ management** | Unfavorable policies around decentralization (cannot make decisions) |  |  |  |  |  | ✓ |  |  |  |  |  |  |
|  | **Public-private partnership** | Private facilities offer more surgical deliveries than public for monetary gains |  |  |  | ✓ |  |  |  |  |  |  |  |  |
|  | **Funding** | No pooling of funds / fund generation at community level |  |  |  |  |  |  |  |  |  |  | A |  |
|  |  | No revolving/ emergency fund at district/upazila level for maintenance |  |  |  |  |  |  |  | ✓ |  |  |  |  |
|  | **Financial barriers to care** | High cost of services/blood products/CS kit |  | ✓ |  |  |  |  |  |  |  |  |  |  |
| Essential Medical Products and Technologies | **Procurement and supply management** | Equipment is not regularly being replaced |  |  |  |  |  |  |  |  |  |  |  | ✓ |
|  |  | Absence of proper inventory system |  |  |  |  |  |  |  | ✓ |  |  |  |  |
| Health Service Delivery | **Service availability / capacity of services** | System for supply of blood / blood products does not meet demand - 24/7 blood transfusion is not always available in all facilities |  |  |  | ✓ |  |  |  | ✓ |  |  |  |  |
|  |  | Insufficient space –theater space |  |  |  |  |  | ✓ |  |  |  |  |  |  |
|  |  | Donor list is not always available in all facilities |  |  |  |  |  |  |  | ✓ |  |  |  |  |
| Health Information System | **Tools for information/ system reporting** | Information system not computerized due to lack of funds and not updated |  |  |  |  |  |  |  |  |  |  | B |  |
|  |  | Non-functioning reporting system for blood transfusion, supplies & reagent and Caesarean section set |  |  |  |  |  |  |  | ✓ |  |  |  |  |
| Community Ownership and Partnership | **Access** | Poor access to blood supply/blood banks |  |  | ✓ |  |  |  |  |  |  |  |  |  |

# D. Table S4: Bottlenecks for quality care during labour and birth for 2 or more interventions

| Healthy System Building Block | Category | Bottlenecks | Africa | | | | | | Asia | | | | | |
| --- | --- | --- | --- | --- | --- | --- | --- | --- | --- | --- | --- | --- | --- | --- |
|  |  |  | Cameroon | DRC | Kenya | Malawi | Nigeria | Uganda | Afghanistan | Bangladesh | India | Nepal | Pakistan | Vietnam |
| Leadership and Governance  Leadership and Governance | **Policy/strategy** | Lack of policy/ enforcement at all levels, particularly for partograph, blood banks and transfusion, blood safety and in disadvantaged areas (SBA, BEmOC, CEmOC) |  | ✓ | ✓ |  | ✓ |  |  |  | A, O |  | A, B, G, K, P | ✓ |
|  |  | Lack of dissemination and implementation of policies including political will and continuity (SBA, CEmOC) |  |  |  |  | ✓ | ✓ |  |  |  | ✓ | K, P |  |
|  |  | Policy is not articulated as rights and gender sensitive (SBA, BEmOC, CEmOC) |  |  |  |  |  |  |  |  | A |  |  |  |
|  |  | No policy/decision for unified Health Management Information Systems nationally (SBA, CEmOC) |  |  |  |  |  |  |  | ✓ |  |  |  |  |
|  | **Guidelines / standards/ protocols** | Lack of national clinical guidelines including emphasis on performing assisted vaginal delivery and CEmOC procedures (SBA, BEmOC, CEmOC) | ✓ |  |  | ✓ |  |  |  | ✓ |  |  | K, P |  |
|  |  | Lack of updated guidelines (BEmOC, CEmOC) |  |  |  |  |  |  | ✓ |  |  |  | S |  |
|  |  | Poor dissemination of Matenral Newborn Child Health standards and guidelines (SBA, CEmOC) | ✓ | ✓ | ✓ |  | ✓ |  |  |  |  |  |  |  |
|  |  | Poor implementation of guidelines (SBA, BEmOC) |  |  | ✓ |  | ✓ |  |  |  |  |  | S |  |
|  |  | No specific standards including for SBAs / trained health worker and quality of care for women (SBA, BEmOC) |  |  |  |  |  |  |  |  |  | ✓ | S | ✓ |
|  |  | Protocol documents are not regularly updated, particularly for caesarean-section (BEmOC, CEmOC) | ✓ |  |  |  |  |  | ✓ |  |  | ✓ | A, P |  |
|  | **Strategy implementation** | BEmOC and CEmOC not given priority as compared to other programs (BEmOC, CEmOC) |  |  |  |  |  |  |  |  |  |  | B, S |  |
| Health Financing  Health Financing | **Funding** | Inadequate funding and ineffective planning for essential supplies and services (SBA, BEmOC, CEmOC) |  | ✓ |  | ✓ | ✓ | ✓ | ✓ |  |  |  | A, B, G, K, P, S | ✓ |
|  |  | Poor allocation, utilization and prioritization of available funds (SBA, BEmOC, CEmOC) |  |  | ✓ |  | ✓ | ✓ |  | ✓ |  | ✓ | A, G, P |  |
|  | **Insurance** | No universal coverage of health insurance / free care for the newborn (SBA, BEmOC) |  |  |  |  |  |  |  |  |  | ✓ |  | ✓ |
|  | **Financial barriers to care** | Financial barriers to scale-up of skilled care (SBA, BEmOC , CEmOC) | ✓ | ✓ |  | ✓ |  |  |  |  |  |  | B, G |  |
|  | **Out-of-pocket expenses / user fees** | Out of pocket expenditure and transport costs for seeking care (SBA, BEmOC, CEmOC) | ✓ | ✓ | ✓ | ✓ | ✓ | ✓ |  | ✓ |  | ✓ | A, B, G, K, S, P |  |
| Health Workforce  Health Workforce | **Number, distribution and role of health workers** | Weak capacity / quality / skills of providers (SBA, BEmOC, CEmOC) | ✓ | ✓ | ✓ | ✓ | ✓ | ✓ | ✓ | ✓ | O | ✓ | A, B, G, K, P, S | ✓ |
|  |  | Inadequate number of providers (e.g. surgeons and anesthesiologists for C-section, obstetricians, lab technicians) (SBA, BEmOC, CEmOC) | ✓ | ✓ | ✓ | ✓ | ✓ | ✓ | ✓ | ✓ | A, O | ✓ | A, B, G, K, P, S | ✓ |
|  |  | Inappropriate allocation/distribution of human resources across facilities (SBA, BEmOC, CEmOC) | ✓ | ✓ | ✓ |  | ✓ | ✓ | ✓ | ✓ |  | ✓ | A, B, G, K, P, S | ✓ |
|  | **Supervision** | Inadequate mentoring and supportive supervision (SBA, BEmOC, CEmOC) | ✓ | ✓ | ✓ | ✓ | ✓ | ✓ | ✓ | ✓ | ✓ | ✓ | ✓ | ✓ |
|  | **Incentives and motivation** | Lack of / limited motivation of staff (SBA, CEmOC) | ✓ |  |  |  | ✓ | ✓ |  | ✓ | A | ✓ |  |  |
|  |  | Lack of incentives and retention e.g. differential salary issues, low wages due to wage bill, lack of performance based management system, career advancement (SBA, BEmOC, CEmOC) |  |  |  |  |  | ✓ |  | ✓ | A, O | ✓ |  |  |
|  | **Training** | Lack of capacity building / competency-based training of health care workers – CME, refresher training / regular updates, career development training, pre-service with particular mention of SBA and partograph (SBA, BEmOC, CEmOC) |  | ✓ | ✓ | ✓ |  |  | ✓ | ✓ | A, O | ✓ | A, B, G, K, P, S | ✓ |
|  |  | Practice manual not available (BEmOC, CEmOC) |  | ✓ |  |  |  |  |  |  |  |  | A, P | ✓ |
|  | **Job descriptions/ aids** | No job description and job aids (BEmOC, CEmOC) | ✓ |  | ✓ |  |  |  |  |  |  | ✓ | B, K, P |  |
| Essential Medical Products and Technologies | **Lack of supplies and equipment** | Inadequate availability of equipment, supplies and drugs (e.g. partographs, blood products, blood banks, surgical kits, anesthesia equipment, drugs, forceps, vacuum extractors, consistent power supply) (SBA, BEmOC, CEmOC) | ✓ | ✓ | ✓ | ✓ |  | ✓ | ✓ | ✓ | A, O | ✓ | A, B  G, K,P | ✓ |
|  | **Procurement and supply management** | Inadequate / inequitable quantification, forecasting, procurement, supply (e.g. lack of reporting on stock out) and distribution of commodities (SBA, BEmOC, CEmOC) | ✓ | ✓ | ✓ | ✓ | ✓ | ✓ | ✓ | ✓ | A, O |  | A, B, G, P |  |
|  |  | No Logistic Management Information Systems (LMIS) in place to assess supplies including oxytocin and vacuum extraction – only demand-based supply (BEmOC, CEmOC) |  |  |  | ✓ |  |  |  | ✓ |  | ✓ | B, K |  |
| Health Service Delivery  Health Service Delivery  Health Service Delivery  Health Service Delivery | **Service availability / capacity of services** | Lack of a sufficient number and distribution of health facilities (SBA, BEmOC) |  |  | ✓ | ✓ | ✓ |  | ✓ | ✓ | O |  | B |  |
|  |  | Lack of basic infrastructures e.g. water, electricity, labor room sanitation (SBA, BEmOC, CEmOC) |  | ✓ | ✓ |  | ✓ | ✓ |  |  | O |  | A, B, K |  |
|  |  | Lack of 24/7 service delivery/skilled attendance (SBA, BEmOC, CEmOC) |  |  | ✓ | ✓ |  | ✓ | ✓ |  | O | ✓ |  |  |
|  |  | 48 hour stay not ensured for many mothers (SBA, BEmOC, CEmOC) |  |  |  |  |  |  |  |  | O |  |  |  |
|  |  | Poor utilization of medicines due to low knowledge, skills, perceptions, motivation, shortage of personnel, time, particularly partograph (SBA, BEmOC, CEmOC) |  |  | ✓ |  | ✓ | ✓ |  |  | O |  | A, B, G, K, S | ✓ |
|  |  | Inadequate capacities of health facility and health post Shuras to perform the expected jobs (SBA, BEmOC, CEmOC) |  |  |  |  |  |  | ✓ |  |  |  |  |  |
|  | **Referrals** | Lack of /non-functional/weak referral system (SBA, BEmOC, CEmOC) |  | ✓ | ✓ | ✓ | ✓ | ✓ | ✓ | ✓ |  | ✓ | B, K, G, P | ✓ |
|  |  | Referral institutions are not fully equipped to handle complications (SBA, BEmOC, CEmOC) |  |  |  |  |  |  |  |  | A |  |  |  |
|  |  | Availability of transportation for timely referral including ambulance service not fuctional and effective (SBA, BEmOC, CEmOC) |  |  |  |  |  |  |  |  |  | ✓ | A, K |  |
|  | **Coverage** | Low/poor coverage (SBA, BEmOC, CEmOC) |  |  |  |  |  |  |  |  |  |  | K, P, S |  |
|  | **Quality of care/ quality improvement** | The quality of services is inadequate/poor (SBA, BEmOC) | ✓ |  |  |  | ✓ |  |  |  |  |  | A, K, P, S |  |
|  |  | Implementation of quality of care is not taking place due to lack of enforcement (SBA, CEmOC) |  | ✓ |  |  | ✓ |  |  |  |  |  |  |  |
|  |  | No monitoring mechanism / Monitoring &Evaluation in place to ensure quality and adherence – lack of institutional quality improvement systems including clinical reviews and audits, performance quality assurance system and performance based feedback (SBA, BEmOC, CEmOC) | ✓ | ✓ |  | ✓ |  | ✓ | ✓ | ✓ |  | ✓ | A, B, G, K, P, S | ✓ |
|  |  | Incorrect and ineffective use of partograph (SBA, CEmOC) |  |  |  | ✓ |  | ✓ |  |  |  |  |  |  |
|  |  | Insufficient dissemination of protocols, particularly on quality improvement (SBA, BEmOC, CEmOC) | ✓ | ✓ | ✓ |  | ✓ |  |  |  |  | ✓ |  |  |
|  | **Communication and health worker attitudes** | Limited communication skills of the health communicators (SBA, BEmOC, CEmOC) |  |  |  |  |  |  |  |  |  |  | A, S | ✓ |
|  | **Private sector** | Lack of private sector involvement (SBA, BEmOC, CEmOC) |  |  |  |  |  |  |  |  | A |  |  |  |
| Health Information System  Health Information System | **Policy** | No policy/decision for unified Health Management Information System (HMIS) nationally (SBA, CEmOC) |  |  |  |  |  |  |  | ✓ |  |  | G |  |
|  | **Tools for information system/ reporting** | Too many logbooks, forms, records, overload to health workers, leading to inaccurate data reporting and collection (SBA, BEmOC. CEmOC) |  | ✓ |  |  |  |  |  |  | O |  | S | ✓ |
|  |  | Data regarding use of partograph not available (SBA, BEmOC, CEmOC) |  |  |  |  |  |  |  |  | O |  |  |  |
|  |  | No central register in facilities (SBA, BEmOC, CEmOC) |  |  |  |  |  |  |  |  | A |  |  |  |
|  | **Data quality** | Facilities records not complete and of poor quality (SBA, BEmOC) | ✓ |  |  |  |  |  |  | ✓ | O |  | S |  |
|  |  | Concerns over completeness and quality of Health Management Information System data (SBA, CEmOC) |  |  | ✓ | ✓ | ✓ |  |  |  |  |  |  |  |
|  | **Private sector** | Private hospitals not reporting on important data (SBA, BEmOC, CEmOC) |  |  |  |  | ✓ | ✓ | ✓ |  | O |  |  |  |
|  | **Indicators** | Incomplete / inconsistent data/indicators at state and national level and by private and public providers at community and facility levels including case fatality rate, skilled care at birth / assisted delivery, EmO(N)C and caesarean-section, blood transfusion, use of oxytocin (SBA, BEmOC, CEmOC) | ✓ | ✓ | ✓ |  | ✓ |  | ✓ | ✓ | A, O |  | A, B, G, K, P | ✓ |
|  |  | Institutional delivery is being reported by default as skilled care (SBA, BEmOC, CEmOC) |  |  |  |  |  |  |  |  | A |  |  |  |
| Health Information System | **Use and dissemination of information** | The use of information is not uniform across all levels (SBA, CEmOC) |  |  |  |  | ✓ |  | ✓ |  |  |  |  |  |
|  |  | Inappropriate capacity of staff in utilization, data collection and analysis of the tools – reasons include: lack of time, overload, too much overlapping records such as partograph and individual medical record (SBA, BEmOC, CEmOC) |  | ✓ | ✓ |  |  | ✓ | ✓ | ✓ | O | ✓ | A, K, S | ✓ |
|  | **Quality assessment system** | Lack of effective system/data to monitor and evaluate the quality of care e.g. review of quality of caesarean sections and assisted vaginal deliveries, maternal, neonatal and neonatal death audits and reviews (BEmOC, CEmOC) | ✓ | ✓ | ✓ |  | ✓ |  | ✓ | ✓ |  | ✓ | B, S | ✓ |
| Community Ownership and Partnership  Community Ownership and Partnership  Community Ownership and Partnership | **Promotion/ communication** | Low promotion/advocacy of skilled care at birth and issues affecting women and newborns (SBA, BEmOC, CEmOC) |  | ✓ | ✓ |  |  |  |  |  |  | ✓ | G, S | ✓ |
|  |  | Overall Information Education Communication materials inadequate and not in local language (SBA, BEmOC, CEmOC) |  |  |  |  |  |  |  | ✓ | A |  | A, P | ✓ |
|  |  | Inadequate communication materials and capacities in their proper usage (SBA, BEmOC, CEmOC) |  |  | ✓ |  |  |  | ✓ |  |  |  |  |  |
|  | **Awareness** | Poor public awareness/knowledge on health seeking, services available and women’s rights in communities (SBA, BEmOC, CEmOC) |  |  |  | ✓ |  | ✓ |  | ✓ | O | ✓ | A, B, G, K | ✓ |
|  | **Care-seeking** | Transport issues especially in difficult terrains and poorest communities (e.g. non-functional ambulance services) (SBA, BEmOC) | ✓ | ✓ |  |  |  |  |  |  |  | ✓ |  | ✓ |
|  | **Barriers / challenges faced by mothers** | Socio-cultural barriers (e.g. gender inequality / low status of women in some communities / lack of empowerment, absence of decision-making and financial authority of women, cultural norms of negative practices in caring for women and children, fatalism) (SBA, BEmOC, CEmOC) | ✓ |  | ✓ | ✓ | ✓ |  | ✓ | ✓ | O |  | B, P | ✓ |
|  |  | High fees for care especially for poor families (SBA, BEmOC, CEmOC) |  | ✓ |  |  |  |  |  |  |  |  |  |  |
|  |  | Majority of women are illiterate (SBA, BEmOC, CEmOC) |  | ✓ |  |  |  |  |  |  |  |  |  |  |
|  | **Access** | Difficult access in relation to the distance between the structures, geographical terrain, cost/Socioeconomic status, equipment, transport, and material for the majority of health facilities (SBA, BEmOC, CEmOC) | ✓ | ✓ | ✓ | ✓ |  | ✓ |  | ✓ | O |  | A, B, K, G, P, S |  |
|  | **Community involvement and mobilization** | Low community involvement – community support groups and public reps not fully active and motivated, lack of motivation, poor ownership and empowerment, public has no say on affairs and policymaking (SBA, BEmOC, CEmOC) | ✓ | ✓ | ✓ | ✓ |  |  |  | ✓ | A |  | A, B, G, K, P, S |  |
|  |  | Low male involvement (SBA, BEmOC, CEmOC) |  | ✓ | ✓ | ✓ | ✓ | ✓ |  |  |  |  |  | ✓ |
|  |  | Community based structures are weak/ not fully functional (SBA, BEmOC, CEmOC) |  |  |  |  | ✓ |  |  |  |  |  | G, P |  |

# E. Table S5: Solutions for quality care during labour and birth for SBA

| SBA | | | | | | |
| --- | --- | --- | --- | --- | --- | --- |
| Health System Building Block | Africa | | | | | |
|  | Cameroon | DRC | Kenya | Malawi | Nigeria | Uganda |
| Leadership and Governance | - Review national standardized protocols, validate, disseminate and to monitor compliance | - Disseminate normative documents to all especially at subnational level - Make available the partograph and guide on use | - Soft copy of guidelines and protocols such as partograph to reach all facilities – to reduce paperwork - Support the RH coordinators who receive the soft copies to distribute - DHMTs carry out supervision to all facilities – could be used for dissemination | - No solutions proposed | - Advocate for rationalization of staff deployment at national and state level based on equity - Implement integrated supportive supervision and mentorship at all levels - Extend supportive supervision and oversight to private sector providers in the health care system to be able to monitor standard of practice and enforce quality of care | - No solutions proposed |
| Health Financing | - Advocacy for reducing financial barriers by grant mechanisms - Scale up of subsidized obstetrical kits - Linking the use of partograph to obtaining funding | - Insurance scheme for skilled care at delivery by the State | - Advocate for Universal health care and social protection policies - Scale up and target the indigents | - No solutions proposed | - Advocate for adoption of health bill currently in Parliament through advocacy - Advocate for women empowerment | - Bring services nearer to the people - Implement income-generating activities and community financing for health (SACCO for health) |
| Health Workforce | - Ensure the recruitment and deployment in priority areas of midwives in training - Strengthen internal and external supervision - Identify a monitoring strategy to fill the partograph for every birth | - Capacity building of staff in emergency obstetric care base - Redeployment of staff trained in the country - Revitalizing the monitoring and supervision | Ensure that essential newborn care is included in the pre-service syllabus | - No solutions proposed | - Strengthening integrated supportive supervision and mentoring - Advocate for additional recruitment and rational deployment of health workers - Reimbursement vouchers for emergency referrals | - Invest in staffing |
| Essential Medical Products and Technologies | - No solutions proposed | - Standardization of the partograph at all levels - Improving the supply and distribution system of the partograph | - No solutions proposed | - No solutions proposed | - Build capacity for procurement, storage and distribution of medical supplies and other related commodities including partograph | - Equip health facilities |
| Health Service Delivery | - Create a demand for quality and enforce quality standards - Implement the accountability | - Improved coverage of skilled care at childbirth - Make available water and electricity - Improve institutional capacity | - No solutions proposed | - No solutions proposed | - Encourage greater private sector participation and involvement in health sector programming including supportive supervision - Strengthen supportive supervision and mentoring - Advocate for infrastructure improvement, deployment of additional resources - Strengthen referral system by institutionalizing the system involving National Union of Road Transport Workers, telecommunication | - No solutions proposed |
| Health Information System | - Restructure the system of health information - Include the number of partograph use by childbirth routine - Assessing the quality of filling programs through periodic surveys | - Complete the flagship indicators related to skilled birth | - No solutions proposed | - No solutions proposed | - Institutionalize Data Quality Assurance - Use of technology in data management - Strengthen inclusion of Community Based Data into routine HMIS | - No solutions proposed |
| Community Ownership and Partnership | - No solutions proposed | - IEC and behavior change (CC) - Revitalizing the work of the relays on the ground | - Respectful care to address the attitude of health workers | - No solutions proposed | - Strengthen coordinated implementation of community health strategy - Integration of community interventions | - Provide education for the mothers in the community during antenatal care - Empower midwives with skills - Empower men to be more involved |

| SBA | | | | | | |
| --- | --- | --- | --- | --- | --- | --- |
| Health System Building Block | Asia | | | | | |
|  | Afghanistan | Bangladesh | India: AP | India: Odisha | Nepal | Vietnam |
| Leadership and Governance | - No solutions proposed | - Short, intermediate and long term human resource plan and strategy for skilled birth attendant at facility and community should be developed based on APR Benchmark - Target fixation for Institutional Birth and Delivery by C-SBA based on local demography, communication, capacity and readiness of the facility and providers - In-depth review of midwifery training plan & strategy including capacity and number of training institutions and facilities - Accreditation system for private midwives | - Establishing at-least one centre of excellence based on the practice benchmarks and not only infrastructure - Spear head and strengthen medical college also link the skill labs initiative - Birth Companion policy and proposal for “mata-shishvula hakkulu” charter to be discussed at the state - Revamping of training planning based on the new guidelines | - All ANMs to be trained on SBA (already in plan) - Special incentives for ANMs placed in difficult and hard to reach areas (V3 and V4 as per vulnerability ranking) - DP expansion across state esp L1 - Improved infrastructure with regard to building, electricity and water - TBAs and private hospitals to be circulated SBA guidelines - Special Incentives on home SBAs utilizing partograph - Policy to be outlined to attract and retain Human Resources | - Capacity building of HR according to their role and responsibilities - Develop and implement strictly performance based evaluation system - Adequate budget allocation for development and strengthens the infrastructure - Review existing sanction post and Increase as need based | - Need to specify the criteria, in order to collect accurate data for report - In order to resolve the fact of filling out the records without practical use of the records, - MoH need to reconsider and give clear guidance for implementation to the lower levels |
| Health Financing | - No solutions proposed | - Re-calibration of the DSF programme and test two models - DSF with Pay for Quality Performance and Only Pay for Quality Performance to providers tested through ‘Pay for Performance ’ | - Improve utilization | - No solutions proposed | - Develop system for free service for newborn - Appropriate and adequate budget allocation for newborn care | - No solutions proposed |
| Health Workforce | - No solutions proposed | - Develop short, intermediate and long term human resources plan and strategy for skilled birth attendance at facility and community levels based on APR benchmark - Revise the policy for enrolment in 6 month Nurse Midwives Training Course and entry into government service as midwives so that private nurses are selected for the course and can join government jobs like midwives - Institutionalize incentive for rural based skilled providers (hardship allowances, P4P, career growth) - Map available skilled birth attendants and ensure equitable deployment - Institutionalize refresher and on job training and mentoring by gov’t & non-gov’t institutions | - Policy initiative to incentivize the services –GoI endorsement needed - Development of pre-service curricula and inclusion in the medical/ nursing government & private regulation from University of Health Sciences - Uniform and career development plan and hard allowances _HR committee - Pool of mentors to monitor the skill labs for all technical elements with realistic plan - Systematic assessment of use of ICT for mentoring and off-site support | - Incentives to be extended to MOS staying in Difficult areas - Medical college staff nurses to be included in the training programme - DP mentoring guidelines finalised, mentors identified, approval received in PIP, training and implementation planned - Nurse practitioners to be included for BEMOC - BEMOC to be included in pre-service training for GNMs and in-service training for ANMs and GNMs - Districts having more vacancies to come up with some attractive schemes to attract and retain HR - More specialist positions created and attractive incentives a/c to place and position - Free food and compensation can be given to attendants for BPL families | - Qualified and skill competent supervisor placement at District, Regional and Centre level - Strengthen the quality training monitoring system - Develop on site coaching plan and refresher training for SBAs - Develop professional Midwife cadres - Develop a system for coaching and mentoring for use of partograph - Take a responsibility for use of partograph by facility in charge | - To strengthen re-training and couching for skills, competency-based training |
| Essential Medical Products and Technologies | - No solutions proposed | - Engaging professional societies (OGSB & BPS) and their subnational units to orient and ensure use of partograph by obstetrician and other SBAs - Institutionalize the culture of supportive supervision and involve elected representative and Civil Society groups in MNCH monitoring - Revise OP-MNC&AH to include funding for partograph printing (RPA/ DPA) | - Directorate to Link with APMSIDC (AP medical services and infrastructure development corporation) to work on EML - Logistic Management system – real time being planned - Centralized essential commodities eg Blood data server | - Sensitisation of HPs and their strict monitoring including prescription audit - Better streamlining of supply chain management - Instruments to be included in ODMIS - Universal implementation of MNH standards with regards to procurement of instrument as per case load - Clear Local purchase guidelines for essential drugs and supplies - Promotion and organisation of voluntary blood donation camp | - No solutions proposed | - No solutions proposed |
| Health Service Delivery | - No solutions proposed | - Release of HA & FWA from their 3 days fixed duty at CC (CHCP is on board) and ensure more domiciliary visits - Maximize the use of CG & CSG for Community Clinics for increasing awareness and health seeking behaviour - Fund generation by CG & CSG to support transport cost of poorest - DSF for selected remote areas with alternate payment mechanism (mobile/ MOU with ambulance company) - Policy to ensure waiver for poorest in private facilities and strong monitoring by local authorities | - Require a plan to strengthen the referral centres and link it with quality assurance - Incentivization of the service delivery providers trained in special trainings - Inclusion of credit of these trainings in MCI for inclusion in career progression and PG entrance - Plan to be articulate | - Training and mentoring to be strengthened to increase use of partograph - Expansion of 24 x 7 services to difficult and hard to reach areas - Decongestion of L2 and L3 - MDR to be strengthened and PDR to be initiated - Regular clinical audits - More number of ambulances with 102 call centres - Private hospital to report important data - Use of tools to promote practice of skills like WHO safe Birth Checklist - Ensure 48 hour stay for mothers after delivery - Improve infrastructure and quality services in labour room | - Develop need based planning focusing to GESI - Increase enough skilled HR sanction post such as Midwives, SBAs at the facilities | - No solutions proposed |
| Health Information System | - No solutions proposed | - Amend existing monthly EmOC reporting format of HMIS to include number of deliveries by C-SBA at home - M&E Task Group of HPNSDP chaired by additional Secretary should undertake initiative for uniform MNCH HMIS from both DGHS & DGFP - Institutionalize regular capacity building effort for Health Managers & statisticians for analysis of HMIS and interpretation for programmatic action | - MIS wing to be actively involved with state and district program in data entry - Nodal officer to be nominated for coordination - Develop a few robust HIS – especially indicators to track the progress - Clinical audits – new initiative - Mechanism to link this to get individual indicators and accountability framework - Committee overseeing the progress | - DP mentoring to include data quality and quality implementation - HMIS to be revised to include more components like Assisted deliveries and CFRs - Private hospital to report important data - Ensure 100% coverage of review using 16 dash board indicators up to Block level - Use of Standardised formats to be promoted - Automation of FRU Records to be scaled up | - Capacity Building of the existing staff on Data Analysis and use of data findings | - Information to be reported should be simplified |
| Community Ownership and Partnership | - No solutions proposed | - Maximize the use of CG & CSG for Community Clinics and CHW (from GO & NGO) for increasing awareness and health seeking behaviour - Multiple sector involvement and action for women’s empowerment focused IPC, counselling & group meeting by CHW & providers with appropriate job aids - Capacity building of providers and supervisor on IPC & counselling | - Work towards quality of care models - Talli sishula hakku initiative – meeting of providers and mothers/ family support groups – working together - Enabling IEC for all levels | - Expansion of DPs for improving accessibility - Special incentives for community mobilisers and service providers - Focussed BCC activities to be planned in local dialect to improve utilisation in PVTGs - Discussion in community forums | - Generalised social audit and awareness - Orientation to management committee on their role and responsibilities - Involvement of community organization in planning implementation and Monitoring of the program - Make health program transparent | - BCC to be strengthened |

| SBA | | | | | | |
| --- | --- | --- | --- | --- | --- | --- |
| Health System Building Block | Pakistan | | | | | |
|  | AJK | Baluchistan | Gilgit- Paltistan | Khayber Pakhtun | Punjab | Sindh |
| Leadership and Governance | - WHO Guidelines are available for skilled care at birth, - Partograph use is recommended at referral levels in some referral centres | - No solutions proposed | - Stream lining of resources - Strategy of MoH | - Train the service providers on the Guidelines and make sure that the necessary protocols are followed | - No solutions proposed | - Need to specify the criteria, in order to collect accurate data for report - In order to resolve the fact of filling out the records without practical use of the records, - MoH need to reconsider and give clear guidance for implementation to the lower levels |
| Health Financing | - Sufficient resources and mechanisms should be allocated | - No solutions proposed | - No solutions proposed | - Ensure accountability mechanisms and curb under the table payment to the service providers | - No solutions proposed | - No solutions proposed |
| Health Workforce | - Remove barriers to ensure deployment of female skilled care providers in remote areas by offering incentives, provision of free residence, security etc. | - No solutions proposed | - Accountability training | - Develop and implement clear Job descriptions for all tiers of service providers and use these for the purpose of monitoring and supervision | - No solutions proposed | - To strengthen re-training and coaching for skills, - Competency-based training |
| Essential Medical Products and Technologies | - Printing of partograph may be included in printing list at state and district level | - No solutions proposed | - No solutions proposed | - Make policy regarding the use of partograph during child birth and make it freely available | - No solutions proposed | - No solutions proposed |
| Health Service Delivery | - Involve private sector in: Sharing data on key indicators - Adherence to standard protocols / use of partograph - Referral support/ transportation | - No solutions proposed | - No solutions proposed | - Expand the coverage and enhance the quality of services to gain people’s confidence and trust - Overhaul the existing services to make them more user-friendly | - No solutions proposed | - No solutions proposed |
| Health Information System | - Existing DHIS needs revisit - Donor support is needed to operationalize DHIS in all 10 districts in AJK - Sufficient resources should be allocated to improve monitoring system | - No solutions proposed | - No solutions proposed | - No solutions proposed | - No solutions proposed | - Information to be reported should be simplified - Need to integrate data from various programs |
| Community Ownership and Partnership | - Sufficient resources should be allocated for community mobilization and education | - No solutions proposed | - Wide spread health education - Community realization and involvement | - No solutions proposed | - No solutions proposed | - BCC to be strengthened - To improve quality of services for gaining people’s trustful attitude |

# F. Table S6: Solutions for quality care during labour and birth for BEmOC

| BEmOC | | | | | | |
| --- | --- | --- | --- | --- | --- | --- |
| Health System Building Block | Africa | | | | | |
|  | Cameroon | DRC | Kenya | Malawi | Nigeria | Uganda |
| Leadership and Governance | - Train EONC staff - Equipping health facilities | - Make available the extension of normative documents and protocol of care | - Dissemination to target service providers at all levels including L1-3 - Establishment of one MNH implementation plan to include partner resource mapping | - No solutions proposed | - Ensure effective distribution and built capacity for sustainable use of the protocols and guidelines | - No solutions proposed |
| Health Financing | - No solutions proposed | - Subsidies for care - Creation of mutual health | - Advocacy for increased allocation – involvement of CSOs, and fast track the MNCH Bill - At county level, promote evidence based planning and establishment of investment cases for MNCH At national level, monitoring of resources-CAF | - No solutions proposed | - Advocate for predicable disbursement of funding to all levels - Advocate for community based health insurance schemes | - No solutions proposed |
| Health Workforce | - Train, recruit and retain staff | - Staff training in basic emergency obstetric care according to the competency-based approach, insufficient numbers and fair redeployment | - Optimise current workforce thru’ capacity building in MNCH interventions - Strengthen supervision in the MNH implementation plan and in cooperate mentorship programs into current trainings – EmONC, FANC etc - Scale up the “Heshima” project to all counties | - No solutions proposed | - Advocate for rational deployment of staff - Staff motivation | - No solutions proposed |
| Essential Medical Products and Technologies | - No solutions proposed | - Strengthening the national drug supply system | - Managers and key service providers to be trained on forecasting and quantification - Logistic management committees for MNCH to be established at national and county levels | - No solutions proposed | - Build procurement, storage and distribution capacity for medical commodities at state level | - No solutions proposed |
| Health Service Delivery | - No solutions proposed | - Increased coverage in BEmOC - Provision of adequate facilities and structures in materials | - Map BEmONC services and identify gaps for action - Innovative approaches to access (carts, MWHs, boats) services including fleet - Institutionalise SBMR tool in all MNCH interventions | - No solutions proposed | - Competency based training in assisted deliveries - Advocate for more resources to referral system | - No solutions proposed |
| Health Information System | - Integrate assisted deliveries data in the routine health information system (distinguishing qualifications of health care staff who delivers women) - Check the completeness of data on births | - Capacity building of service providers for data collection | - Advocacy for recruitment at national and county levels - Improve capacity of HRIO and health managers to manage data for decision making | - No solutions proposed | - Advocate for increased accountability of private sector in health - Establishment of perinatal death audits as part of MDRs - Strengthen NHMIS including adoption of technology | - No solutions proposed |
| Community Ownership and Partnership | - No solutions proposed | - Community awareness - Involvement of men in the accompaniment to BEmOC | - Expand and strengthen CHS specifically for MNCH - Social mobilisation and community involvement in MNCH activities | - No solutions proposed | - Strengthened community health services linkages as part of community health strategy scale up - Community social mobilization for positive health actions - Women empowerment | - No solutions proposed |

| BEmOC | | | | | | |
| --- | --- | --- | --- | --- | --- | --- |
| Health System Building Block | Asia | | | | | |
|  | Afghanistan | Bangladesh | India: AP | India: Odisha | Nepal | Vietnam |
| Leadership and Governance | - No solutions proposed | - Joint circular and monitoring from DGHS & OGSB - Short, intermediate and long term human resources plan and strategy for skilled birth attendants at facility and community levels should be developed based on APR Benchmark | - Establishing at-least one centre of excellence based on the practice benchmarks and not only infrastructure - Spear head and strengthen medical college - Also link the skill labs initiative - Birth Companion policy and proposal for “mata-shishvula hakkulu” charter to be discussed at the state - Revamping of training planning based on the new guidelines | - All ANMs to be trained on SBA (already in plan) - Special incentives for ANMs placed in difficult and hard to reach areas (V3 and V4 as per vulnerability ranking) - DP expansion across state esp L1 - Improved infrastructure with regard to building, electricity and water - TBAs and private hospitals to be circulated SBA guidelines - Special Incentives on home SBAs utilizing partograph - Policy to be outlined to attract and retain HR | - No solutions proposed | - No solutions proposed |
| Health Financing | - No solutions proposed | - Provision of emergency fund at local level include B-EmOC drugs/supplies in MSR by Civil Surgeons | - Improve utilization | - No solutions proposed | - No solutions proposed | - No solutions proposed |
| Health Workforce | - No solutions proposed | - Develop short, intermediate and long term human resources plan and strategy for Skilled Birth Attendants at facility and community levels - Institutionalize incentives for rural based skilled providers (hardship allowances, P4P, career growth) | - Policy initiative to incentivize the services –GoI endorsement needed - Development of pre-service curricula and inclusion in the medical/ nursing government & private regulation from University of Health Sciences - Uniform and career development plan and hard allowances HR committee - Pool of mentors to monitor with the skill labs for all technical elements with realistic plan - Systematic assessment of use of ICT for mentoring and off-site support | - Incentives to be extended to MOS staying in Difficult areas - Medical college staff nurses to be included in the training programme - DP mentoring guidelines finalised, mentors identified, approval received in PIP, training and implementation planned - Nurse practitioners to be included for BEMOC - BEMOC to be included in pre-service training for GNMs and in-service training for ANMs and GNMs - Districts having more vacancies to come up with some attractive schemes to attract and retain HR - More specialist positions created and attractive incentives according to place and position - Free diet and compensation can be given to attendants for BPL families | - No solutions proposed | To strengthen re-training and couching for skills   - Competency-based training |
| Essential Medical Products and Technologies | - No solutions proposed | - Strengthen the supply and procurement management including web based stock register system - Provision of funds and authority for local purchase through local level Plan | - Directorate to Link with APMSIDC (AP medical services and infrastructure development corporation) to work on EML - Logistic Management system – real time being planned - Centralized essential commodities eg Blood data server | - Sensitisation of HPs and their strict monitoring including prescription audit - Better streamlining of supply chain management - Instruments to be included in ODMIS - Universal implementation of MNH standards with regards to procurement of instrument as per case load - Clear Local purchase guidelines for essential drugs and supplies - Promotion and organisation of voluntary blood donation camp | - No solutions proposed | - No solutions proposed |
| Health Service Delivery | - No solutions proposed | - Joint circular and monitoring from DGHS & OGSB - Mapping of available B-EmOC facilities with signal function (public, NGO & private) and plan to address human resource and equipment needs | - Require a plan to strengthen the referral centres and link it with quality assurance - Incentivization of the service delivery providers trained in special trainings - Inclusion of credit of these trainings in MCI for inclusion in career progression and PG entrance - Plan to be articulate | - Training and mentoring to be strengthened to increase use of partograph - Expansion of 24 x 7 services to difficult and hard to reach areas - Decongestion of L2 and L3 - MDR to be strengthened and PDR to be initiated - Regular clinical audits - More number of ambulances with 102 call centres - Private hospital to report important data - Use of tools to promote practice of skills like WHO safe Birth Checklist - Ensure 48 hour stay for mothers after delivery - Improve infrastructure and quality services in labour room | - No solutions proposed | - No solutions proposed |
| Health Information System | - No solutions proposed | - No solutions proposed | - MIS wing to be actively involved with state and district program in data entry - Nodal officer to be nominated for coordination - Develop a few robust HIS – especially indicators to track the progress - Clinical audits – new initiative - Mechanism to link this to get individual indicators and accountability framework - Committee overseeing the progress | - DP mentoring to include data quality and quality implementation - HMIS to be revised to include more components like Assisted deliveries and CFRs - Private hospital to report important data - Ensure 100% coverage of review using 16 dash board indicators up to Block level - Use of Standardised formats to be promoted - Automation of FRU Records to be scaled up | - No solutions proposed | - Information to be reported should be simplified - Need to integrate data from various programs |
| Community Ownership and Partnership | - No solutions proposed | - No solutions proposed | - Work towards quality of care models - Talli sishula hakku initiative – meeting of providers and mothers/ family support groups – working together - Enabling IEC for all levels | - Expansion of DPs for improving accessibility - Special incentives for community mobilisers and service providers - Focused BCC activities to be planned in local dialect to improve utilisation in PVTGs - Discussion in community forums | - No solutions proposed | - BCC to be strengthened - To improve quality of services for gaining people’s trustful attitude |

| BEmOC | | | | | | |
| --- | --- | --- | --- | --- | --- | --- |
| Health System Building Block | Pakistan | | | | | |
|  | AJK | Baluchistan | Gilgit- Paltistan | Khayber Pakhtun | Punjab | Sindh |
| Leadership and Governance | - System will be developed to ensure that standard protocols and standards are strictly followed - For assisted vaginal delivery RMNCH has a plan/strategy, policies and national standard treatment guideline or clinical protocols - Assisted vaginal delivery as part of BEmOC available at first, second and tertiary level | - No solutions proposed | - Priority setting by MoH | - The Treatment Guidelines and Clinical protocols should be officially adopted and made available to service providers - Make sure that the guidelines are followed | - No solutions proposed | - No solutions proposed |
| Health Financing | - Sufficient funds to be allocated to maintain BEmOC services at First level care facility | - No solutions proposed | - Priority taking - More allocations | - The budget allocation should be made in such a way so that it addresses the coverage and quality issues | - No solutions proposed | - No solutions proposed |
| Health Workforce | - Increase number of skilled birth attendants authorized to prescribe/administer oxytocin - Manuals detailing standards of practice to be provided to all staff - Mechanism required for checking competencies of Health workers providing BEmOC services | - No solutions proposed | - Training and monitoring | - Ensure that all service providers receive competency based pre-service and on-the-job training - The staff placements between urban and rural areas should be equitable and need based - Develop a strong Monitoring and supervisory system in line with the job description of the service providers | - To start and implement training programs - To ensure CMEs | - To strengthen re-training and couching for skills - Competency-based training |
| Essential Medical Products and Technologies | - Vacuum Extractors and forceps to be provided to first level referral facilities providing BEmOC services - Need for functional logistic system to asses adequate supplies of essential drugs | - No solutions proposed | - No solutions proposed | - Revive/strengthen the Logistics Information System to make it more responsive to the logistical needs of all health facilities | - Policy needs to be developed - Constant supply to be ensured | - No solutions proposed |
| Health Service Delivery | - Efforts to be made by all health facilities to promote quality BEmOC Services | - No solutions proposed | - No solutions proposed | - Ensure that facilities offering BEmOC are well equipped, well-staffed and equitably spread between urban and rural areas | - No solutions proposed | - No solutions proposed |
| Health Information System | - Quality HMIS system needs to be strengthened | - No solutions proposed | - Major attention | - Make BEmOC as part of the HMIS - Generate, compile, analyze and use data for evidence based decision making | - System for collecting information | - Information to be reported should be simplified - Need to integrate data from various programs |
| Community Ownership and Partnership | - A functional communication system between Health facility and ambulance (public and private) will be developed | - No solutions proposed | - No solutions proposed | - Take culturally appropriate measures to ensure enhanced and meaningful community participation | - Improve community awareness | - BCC to be strengthened - To improve quality of services for gaining people’s trustful attitude |

# G. Table S7: Solutions for quality care during labour and birth for CEmOC

| CEmOC | | | | | | |
| --- | --- | --- | --- | --- | --- | --- |
| Health System Building Block | Africa | | | | | |
|  | Cameroon | DRC | Kenya | Malawi | Nigeria | Uganda |
| Leadership and Governance | - SRMNI finalize the strategic plan 2014 - 2020 and plan to reduce maternal and infant mortality - Make functional the technical working group on the health of the mother and the newborn | - Advocacy Capacity Building NTSP - Advocacy to make available the CS kit in institutions of care through the national supply system | - Develop MNH implementation Plan to include specific actions for scaling up CEmONC - Develop SOPs and Job Aids/Algorithms for C/S indications and Blood transfusions - Use of Ketamine especially in remote areas | - No solutions proposed | - Update, package, distribute and train on use of approved protocols and guidelines for RMNCH including EmONC | - Support districts to consolidate health teams – perhaps in 1-2 facilities in district with full EmOC - Coordinating body for maternal newborn |
| Health Financing | - Obstetric kits - Health check - Performance-based financing - Support the implementation of the National Program of Blood Transfusion | - Subsidy care by the State - Promotion of mutual health | - Advocate for increased allocation to Health and specifically to have a special MNH allocation through facilitation of the MNCH Bill | - No solutions proposed | - Advocate for strengthening of state hospital management committees / boards to make them more functional - Women empowerment | - No solutions proposed |
| Health Workforce | - Improve motivation of midwives - Motivations to develop through performance based financing | - Training CEmOC staff according to skills-based approach, and sufficient - Creation of favorable working conditions in rural areas for PNDRH - Competency-based training and adequate deployment | - Advocacy for training more anaesthetists + theatre nurses and encourage alternatives to G/A - Spinal anaesthesia - Capacity building to enhance skills of available health work force - Develop clear job description and Job Aids and SOPs - Strengthen supervision and in cooperate mentoring within trainings | - No solutions proposed | - Advocate for additional recruitment and rational distribution of drugs | - Improve wages (or improve morale through recognition of good work, other - Resuscitation- Mentoring, supplies |
| Essential Medical Products and Technologies | - Strengthen the procurement system - Equipping health facilities (CEmONC) | - Ensure regular and adequate supply (normal operation PNAM / NTSP) - Grant of care (blood transfusion and caesarean section) by the State | - Expand services to cover all regions - Enforcement of policy for implementation of Blood safety universally - Capacity building on forecasting and quantification to ensure adequate supplies - Advocacy at county level for adequate procurement - Establish blood banks in every County | - No solutions proposed | - Advocacy for timely release of funds | - No solutions proposed |
| Health Service Delivery | - No solutions proposed | - Subsidize the service by the State - Make available the practice manual at all levels | - To be included in the MNH scale up implementation plan for adequate resource mobilization - Use of innovation for referral services and improve fleet management in public facilities - BCC activities to be enhanced | - No solutions proposed | - Strengthening Public Private Partnerships - Built capacity of existing CEmONC facilities | - No solutions proposed |
| Health Information System | - Strengthen the health information system and evaluation - Conduct periodic BEmONC / CEmONC | - Capacity building of service providers for data collection | - There is need for clear understanding of EmOC related indicators at county and national level - Capacity building on data management at all levels - Review MPDSR system to include quality of C/S and Blood transfusion | - No solutions proposed | - Establish perinatal death audit alongside MDRs - Establish Data Quality Assurance | - No solutions proposed |
| Community Ownership and Partnership | - No solutions proposed | - Community awareness - Male involvement | - Health education and information sharing to be enhanced - Voucher system to target the very poor for provision of transport and referral services and to cover for delivery services in FBO facilities which are currently not covered in the free delivery package - Community engagement and involvement in planning and implementation of CEmONC interventions | - No solutions proposed | - Strengthen implementation of community based strategies and linkages - Community social mobilization and advocacy | - No solutions proposed |

| CEmOC | | | | | | |
| --- | --- | --- | --- | --- | --- | --- |
| Health System Building Block | Asia | | | | | |
|  | Afghanistan | Bangladesh | India: AP | India: Odisha | Nepal | Vietnam |
| Leadership and Governance | - No solutions proposed | - Develop short, intermediate and long term human resources plan and strategy for Skilled Birth Attendants at facility and community levels - Map available C-EmOC facilities and develop 24/7 human resource plan - Institutionalize incentives for rural based skilled providers (hardship allowances, P4P, career growth) - UHFPO/ health manager to conduct blood grouping campaign and donor list with mobile number | - Establishing at-least one centre of the excellence based on the practice benchmarks and not only infrastructure - Spear head and strengthen medical college - Also link the skill labs initiative - Birth Companion policy and proposal for “mata-shishvula hakkulu” charter to be discussed at the state - Revamping of training planning based on the new guidelines | - All ANMs to be trained on SBA (already in plan) - Special incentives for ANMs placed in difficult/hard to reach areas (V3 and V4 as per vulnerability ranking) - DP expansion across state esp L1 - Improved infrastructure - building, electricity and water - TBAs and private hospitals to be circulated SBA guidelines - Special Incentives for SBA utilization of partograph at home - Policy to attract and retain HR | - No solutions proposed | - It is better to concentrate effort to certain districts for being capable of CS and blood transfusion - It is not necessary to ask all of the district hospitals to be capable of CS |
| Health Financing | - No solutions proposed | - DSF with Pay for Quality Performance in selected areas | - Improve utilization | - No solutions proposed | - No solutions proposed | - No solutions proposed |
| Health Workforce | - No solutions proposed | - Develop short, intermediate and long term human resources plan and strategy for Skilled Birth Attendants at facility and community levels - Institutionalize incentives for rural based skilled providers (hardship allowances, P4P, career growth) map available C-EmOC facilities and develop 24/7 human resource plan - Long term plan for ensure at least 3 pairs for C-EmOC facilities | - Policy initiative to incentivize the services –GoI endorsement needed - Development of pre-service curricula and inclusion in the medical/ nursing government & private regulation from University of Health Sciences - Uniform and career development plan and hard allowances HR committee - Pool of mentors to monitor with the skill labs for all technical elements with realistic plan - Systematic assessment of use of ICT for mentoring and off-site support | - Incentives to be extended to MOS staying in Difficult areas - Medical college staff nurses to be included in the training programme - DP mentoring guidelines finalised, mentors identified, approval received in PIP, training and implementation planned - Nurse practitioners to be included for BEMOC - BEMOC to be included in pre-service training for GNMs and in-service training for ANMs and GNMs - Districts having more vacancies to come up with some attractive schemes to attract and retain HR - More specialist positions created and attractive incentives a/c to place and position - Free diet and compensation can be given to attendants for BPL families | - No solutions proposed | - To strengthen re-training and couching for skills - Competency-based training |
| Essential Medical Products and Technologies | - No solutions proposed | - Strengthen the supply and procurement management including web based stock register system - Provision of funds and authority for local purchase through Local level plan | - Directorate to Link with APMSIDC (AP medical services and infrastructure development corporation) to work on EML - Logistic Management system – real time being planned - Centralized essential commodities eg Blood data server | - Sensitisation of HPs and their strict monitoring including prescription audit - Better streamlining of supply chain management - Instruments to be included in ODMIS - Universal implementation of MNH standards with regards to procurement of instrument as per case load - Clear local purchase guidelines for essential drugs and supplies - Promotion and organisation of voluntary blood donation camp | - No solutions proposed | - No solutions proposed |
| Health Service Delivery | - No solutions proposed | - Regular capacity building of District and Upazila Managers on planning and management - Institutionalize effective referral system (Referral Hub, Referral Facilitator at facility level) | - Require a plan to strengthen the referral centres and link it with quality assurance - Incentivization of the service delivery providers trained in special trainings - Inclusion of credit of these trainings in MCI for inclusion in career progression and PG entrance - Plan to be articulate | - Training and mentoring to be strengthened to increase use of partograph - Expansion of 24 x 7 services to difficult and hard to reach areas - Decongestion of L2 and L3 - MDR to be strengthened and PDR to be initiated - Regular clinical audits - More number of ambulances with 102 call centres - Private hospital to report important data - Use of tools to promote practice of skills like WHO safe Birth Checklist - Ensure 48 hour stay for mothers after delivery - Improve infrastructure and quality services in labour room | - No solutions proposed | - No solutions proposed |
| Health Information System | - No solutions proposed | - Quarterly spot check as sample basis to cross check indication for C/section - M&E Task Group of HPNSDP chaired by additional Secretary should undertake initiative for uniform MNCH HMIS from both DGHS & DGFP - Institutionalize regular capacity building effort for Health Managers & statisticians for analysis of HMIS and interpretation for Programmatic action | - MIS wing to be actively involved with state and district program in data entry - Nodal officer to be nominated for coordination - Develop a few robust HIS – especially indicators to track the progress - Clinical audits – new initiative - Mechanism to link this to get individual indicators and accountability framework - Committee overseeing the progress | - DP mentoring to include data quality and quality implementation - HMIS to be revised to include more components like Assisted deliveries and CFRs - Private hospital to report important data - Ensure 100% coverage of review using 16 dash board indicators up to Block level - Use of Standardised formats to be promoted - Automation of FRU Records to be scaled up | - No solutions proposed | - Information to be reported should be simplified - Need to integrate data from various programs |
| Community Ownership and Partnership | - No solutions proposed | - Focused IPC, counselling & group meeting by CHW & providers with appropriate job aids - Capacity building of providers and supervisor on IPC & counselling - Maximize the use of CG & CSG for Community Clinics and CHW (from GO & NGO) for increasing awareness and health seeking behaviour - Women’s empowerment through multiple sector involvement and action | - Work towards quality of care models - Talli sishula hakku initiative – meeting of providers and mothers/ family support groups – working together - Enabling IEC for all levels | - Expansion of DPs for improving accessibility - Special incentives for community mobilisers and service providers - Focussed BCC activities to be planned in local dialect to improve utilisation in PVTGs - Discussion in community forums | - No solutions proposed | - BCC to be strengthened - To improve quality of services for gaining people’s trustful attitude |

| CEmOC | | | | | | |
| --- | --- | --- | --- | --- | --- | --- |
| Health System Building Block | Pakistan | | | | | |
|  | AJK | Baluchistan | Gilgit- Paltistan | Khayber Pakhtun | Punjab | Sindh |
| Leadership and Governance | - No solutions proposed | - No solutions proposed | - Realization, commitment and priority setting | - CEmOC services should be made available through a need based and equitable geographic spread - The Treatment Guidelines and Clinical protocols should be officially adopted and made available to service providers - Make sure that the guidelines are followed | - No solutions proposed | - No solutions proposed |
| Health Financing | - Sufficient funds to be allocated by Government - Donor funding may be sought - Philanthropists may be approached - Public private partnership | - No solutions proposed | - Major allocations | - The budget allocation should be made in such a way so that it addresses the coverage and quality issues surrounding CEmOC | - No solutions proposed | - No solutions proposed |
| Health Workforce | - Ensure supervision and mentoring mechanism for all health workers that provide CEmOC services maintain their competency to perform caesarean sections and blood as per national guidelines - All necessary workforce needs to be provided, options suggested are: Task shifting Delegation of responsibilities, PG Rotation - Payment of additional incentives to staff working in far Flung areas | - No solutions proposed | - Training and incentive for Obstetricians, anaesthetics, operating theatre technician | - Ensure that all service providers receive competency based pre-service and on-the-job training - The staff placements between urban and rural areas should be equitable and need based - Develop a strong Monitoring and supervisory system in line with the job | - No solutions proposed | - No solutions proposed |
| Essential Medical Products and Technologies | - Forecasting should be ensured | - No solutions proposed | - No solutions proposed | - Ensure availability of blood/blood products and other essential equipment and drugs - Revive/strengthen the Logistics Information System to make it more responsive to the logistical needs of all health facilities | - No solutions proposed | - No solutions proposed |
| Health Service Delivery | - Clinical audits and managerial issues need to be sorted and implemented at all levels | - No solutions proposed | - No solutions proposed | - Ensure that facilities offering CEmOC are well equipped, well-staffed and equitably spread between urban and rural areas | - No solutions proposed | - No solutions proposed |
| Health Information System | - There is a need to update the DHIS and Data collection needs to include information on blood transfusion and case fatality rates - Indicators to monitor CEmONC facilities need to be collected continuously and data analysis needs - Improvement with timely reliable data sharing is ensured in order to improve performances - Regular audits should be done | - No solutions proposed | - No solutions proposed | - Ensure that CEmOC is part of the HMIS - Collect and analyse, and use data for quality improvement and informed decision making | - No solutions proposed | - No solutions proposed |
| Community Ownership and Partnership | - Community involvement is critical and should be ensured at all levels | - No solutions proposed | - No solutions proposed | - No solutions proposed | - No solutions proposed | - No solutions proposed |

# H. Table S8: Bottleneck themes and solutions identified for SBA, BEmOC and CEmOC

| Healthy System Building Block | Solutions / Strategies identified by countries teams for each health system building block | | | Themes |
| --- | --- | --- | --- | --- |
|  | SBA | BEmOC | CEmOC |  |
| **Leadership and Governance** | National authorities should be more proactive to implement (develop, train, disseminate to all levels) evidence based standards including for private facilities and facilitate regular mentoring and supervision for SBA, develop appropriate strategies to promote skilled care at birth.  Nigeria: Advocacy for rational deployment of staff and strengthen existing integrated supportive supervision mechanisms.  For India: State level Birth companion policy. | Same as SBA across all countries.  For Kenya: Develop a unified national implementation plan for MNH (covers MoH and partners)  For Bangladesh: Joint monitoring with Obstetricians and DoH. | Develop SOPs, Job Aids, Algorithms for C/S and blood transfusions, National Implementation plan, Functional TWG overseeing implementation  Kenya: Permissive policy for task shifting E.g.: Anaesthetist assistants could use ketamine.    For Pakistan: Planning for CEmOC services should be based on geography, unmet need and ensure equitable services. | **For all:**  National policies support the use of Evidence based Rx Guidelines Supportive supervision For CEmOC: Advocacy and coordination |
| **Health Financing** | Advocacy to increase the financial envelope for MNH to remove financial barriers to care seeking. Ensure that in-built mechanism exist to minimise misuse and corruption of financial resources (Pakistan: Bribes/under the table).  Innovative mechanisms could include Universal Health Coverage, Health insurance (DRC), social protection schemes (Kenya), Results Based Financing (linkage with partograph use- Cameroon).  Bangladesh: testing of new P4P models, Inclusion of newborn care into free care for mothers and children, Community groups to raise funds. Pro- poor policies and fee exemptions.  Pakistan: Financing for community level activities. | Ensure funds for BEmOC service expansion including at primary health facility level. Prioritise resource allocation for MNH, Remove financial barriers, (subsidies DRC, MNCH bill and investment case- Kenya, CBHI- Nigeria)  Bangladesh: Availability of emergency funding at local level.  Nigeria: Ensure multiyear predictable financing for MNH. | For all: Support CEmOC service expansion through in-country and external resources, Innovative financing, remove of any financial barriers, increase resource envelope. Financing for blood bank (Cameroon)  Nigeria: Decentralise (ensure accountability) and strengthen hospital management committees. Planning and budget allocation should be done based on actual need across different contexts. | Removal of financial barriers, Advocacy for increased resources |
| **Health Workforce** | For all countries: Prioritise investment for training health workforce, adequate recruitment, rational deployment and redeployment, ongoing retention and capacity building. Clear job descriptions. Supportive supervision and mentoring. Competency based training curriculum should be developed for SBA and also included in pre-service curriculum for doctors and nurses.  For Pakistan: provision of appropriate incentives and guarantee safety of female health workers esp. in remote areas.  For Cameroon: Monitor routine partograph use through innovative mechanisms.  Kenya: NBC should be included in pre-service curriculum of SBAs.  For Bangladesh: Need a HR plan with short, medium and intermediate targets For Bangladesh: develop accreditation system for SBAs (e.g.: private midwives), involve private sector health workers such as nurses in SBA trainings and create positions for them in public sector.  For Nepal: Increase the sanctioned posts within the public sector based on actual need, Quality assurance of trainings, On-site trainings and refreshers, Develop professional cadres of midwives. System for coaching and mentoring on partograph use.  India: Involvement of academic institutions in pre-service curriculum development and establish a pool of national master trainers who provided ongoing mentorship esp. for skills development. Use of ICT in training, Accreditation systems for health workers. | Same as SBA across all countries. Focus on competency based trainings, refreshers and CMEs. Rational deployment of staff so that staff distribution is equitable and needs based.  For Pakistan: Scale up SBA production and reform policy to administration of Oxytocin.  For Kenya: Heshima project offers a good example of promotion of respectful care at facilities. Ensure staff motivation (salaries, hardship allowances, career ladder, and supervision, regular on the job trainings, appreciation and positive feedback).  Bangladesh: Mapping of HR or establishment of a Human resource information system.  India: Use ICT in trainings. For India: Nurse practitioners to be included for BEmOC, BEmOC to be included in pre-service and in-service training for ANMs and GNM. Recruitment and Retention of specialists. More specialist posts created, improve working conditions. Accreditation systems for health workers. | For all countries: Competency based trainings, implement SOPs, develop job aids, improvement of overall work climate esp. in rural areas and ensure staff motivation (salaries, career ladder, supervision, regular on the job trainings, appreciation and positive feedback).  For Cameroon: rewarding good performance and improving QoC through performance based incentives.  For Kenya: Task shifting for anaesthetists (permissive policy for using A/A for spinal rather than GA);  India: Use ICT in trainings. Accreditation systems for health workers.  Pakistan: staff rotation, supportive supervision. | Competency based Training, HW Motivation, Appropriate human resource management including work shifts and rotation of staff, Incentives and motivation including supportive supervision. |
| **Essential Medical Products and Technologies** | For all countries: Strengthen logistics management and supply of essential commodities including partograph (printing at local levels), Strengthen health facilities.  For Bangladesh: Include partogram and paper in budget of existing national plans.  For India: commodities to be used during labour and delivery to be included in essential drugs list. Real time LMIS to be established, Centralised blood data storage to be instituted, Prescription audits, Clear guidelines for local procurement of Essential drugs and supplies. Blood donation camps | For all countries: Strengthen procurement and logistics management and supply of essential commodities for BEmOC, Decentralization and purchasing systems at the local level.  Kenya: MNCH committee, which oversees logistics management.  Nigeria: Capacity building at state level for procurement, storage and distribution.  For Bangladesh: Web based stock register system.  For India: commodities to be used during labour and delivery to be included in essential drugs list. Real time LMIS to be established, Centralised blood data storage to be instituted, Prescription audits, Clear guidelines for local procurement of Essential drugs and supplies. Blood donation camps  For Pakistan: Vacuum extractors and forceps to be made available at first level facilities | For all countries: Logistics management capacity strengthening, improve health facility infrastructure and capacity. Ensure availability and expansion of blood transfusion and C/S services across the country.  For Kenya: expand C/S services across the country, establish blood banks in every county. DRC: provision of C/S kits and blood transfusion free of charge.  For India: commodities to be used during labour and delivery to be included in essential drugs list. Real time LMIS to be established, Centralised blood data storage to be instituted, Prescription audits, Clear guidelines for local procurement of Essential drugs and supplies. Blood donation camps | Logistics management capacity building, Infrastructure strengthening and expanding EmOC services. |
| **Health Service Delivery** | For all countries: Improve facility based QoC during L& D, Quality assurance, Clinical audits, ensure that HFs are functional, Strengthen accountability, Supportive supervision and monitoring. Expansion of 24/7 services esp. in hard to reach areas. Referral support in case of emergencies.   For NG: Strengthen referral systems for complicated pregnancies by involving other sectors (Roads, transport, telecommunications).  For Bangladesh: Estimate no. of cases that come to a C-SBA, or institution so that standards can be met.  For India: Develop centre for excellence and link in service trainings with skills lab, incentivise partograph use at home births by SBAs.  For Bangladesh: Orient practitioners on partograph use and evidence based standards  India: Decongestion of level 2 and level 3, Maternal Death Reviews to be strengthened and Perinatal death reviews to be initiated, Increase numbers of ambulances and call centres. Data from the private sector to be involved.  Nepal: Context specific planning to ensure equity and social inclusion. For Pakistan: Ensure that services are User friendly. | For all countries: Improve facility based QoC during L& D, Institutionalise clinical audits, ensure that HFs are functional, Quality assurance, accountability. Expansion of 24/7 services esp. in hard to reach areas. Equity in health service delivery and planning for services.  For Kenya: Mapping of BEmOC sites to identify coverage gaps, Improve access to services through innovations such as maternity waiting homes, boats, carts, ambulance fleets.  India: Decongestion of level 2 and level 3, Maternal Death Reviews to be strengthened and Perinatal death reviews to be initiated, Increase numbers of ambulances and call centres.  For Bangladesh: Mapping of BEmoc facilities and creation of plans to address HR needs.  For e.g.: Pakistan: Ensure that assisted vaginal delivery services are available at all levels. | For all countries: QoC improvement, ensure 24/7 CEmoC functionality, improve referral systems. Quality assurance.  For Kenya: awareness creation activities at CEmOC sites.  NG: Public private partnerships for CEmOC services.  India: Decongestion of level 2 and level 3, Maternal Death Reviews to be strengthened and Perinatal death reviews to be initiated, Increase numbers of ambulances and call centres.  For Bangladesh: Capacity building of district managers, Creation of referral hubs and referral facilitators. | QoC, Facility infrastructure, strengthen referral linkages. |
| **Health Information System** | For all countries: Strengthen national HMIS and routine monitoring of programmes, Establish high level oversight mechanism for HMIS, data quality assurance and build national capacity for using data for decision making. Standardize and simplify HMIS.  For NG: include community based data into routine HMIS, mobile technologies for data capture and management.  For India: MIS systems to be developed and indicators added to HMIS. India: Central level MIS to be linked with state level MIS, Focal persons needed for MIS, Standardize indicators for Maternal and newborn health, Integrate data from private hospitals, Use standard indicators to monitor performance at the block level (16 dashboard indicators), Automation of FRU records, Include clinical audit information in MIS,  Bangladesh: Amend existing EmoC forms for HMIS to include care provided by C-SBAs. High level task force to be established.  For Pakistan: Additional donor supply for DHIS needed in 10 districts in AJK. | For all countries: Build national capacity for data driven decision making, strengthen vital registration systems and also national HMIS.  For NG: Integrate private sector data into HMIS, include perinatal deaths into existing maternal death reviews, and strengthen HMIS through technological advances.  For Cameroon: disaggregated data on type of delivery and health worker.  For Pakistan: BEmOC needs to be a part of HMIS. | For all countries: strengthen HMIS, data quality assurance, regular monitoring of functionality of CEmOC indicators, Regular reporting of availability of blood transfusion and case fatality rates. Capacity building for data management, High level oversight committee, Integration of data, Ensure CEmOC is a part of the HMIS,  For Kenya: training on standard monitoring indicators for EmOC.  For NG: Perinatal deaths to be included in the existing audits.  For Bangladesh: Quarterly spot check to see whether indications for C/S were followed. | HMIS strengthening, Data quality assurance, Capacity building for data driven decision making, Integration, simplification of HIMS, Standardized indicators. |
| **Community Ownership and Partnership** | For all countries: Health education, promotion and demand creation for improved care seeking for SBA, Promotion of transparency and social accountability, capacity building of Frontline workers, Community partnership.  For e.g.: Talli Sishula hakku initiative in India, Special incentives for community mobilisers, volunteers, and context specific IEC tools needed.  Kenya: promote respectful care,  NG: integrate community based health programmes,  For UGA: promote male involvement in labour and delivery.  For Bangladesh: Maximise use of community groups and community support groups to increase awareness and health seeking behaviour. Involve multiple sectors for women's empowerment, Focussed IPC, counselling and group meetings.  For Nepal: Community/ Social audits, orientation to HFOMC, involvement in planning. | For all countries: Community engagement and mobilization, strengthen continuum of care from households to health facilities. Same as SBA.  For DRC: Male involvement.  For Pakistan: A functional communication system between facilities and ambulance services (both public and private facilities) needs to be developed. | For all countries: Health education, promotion, social mobilization and strengthening referral linkages, Job aids, Capacity building of providers and counsellors,  For Kenya: community involvement in planning of CEmOC services. | Health promotion, Education, community engagement. Male involvement, referral linkages, Promote transparency and accountability towards communities. |

# I. Figure S1: Subnational grading of bottlenecks for quality care during labour and birth for SBA, BEmOC and CEmOC

**SBA**


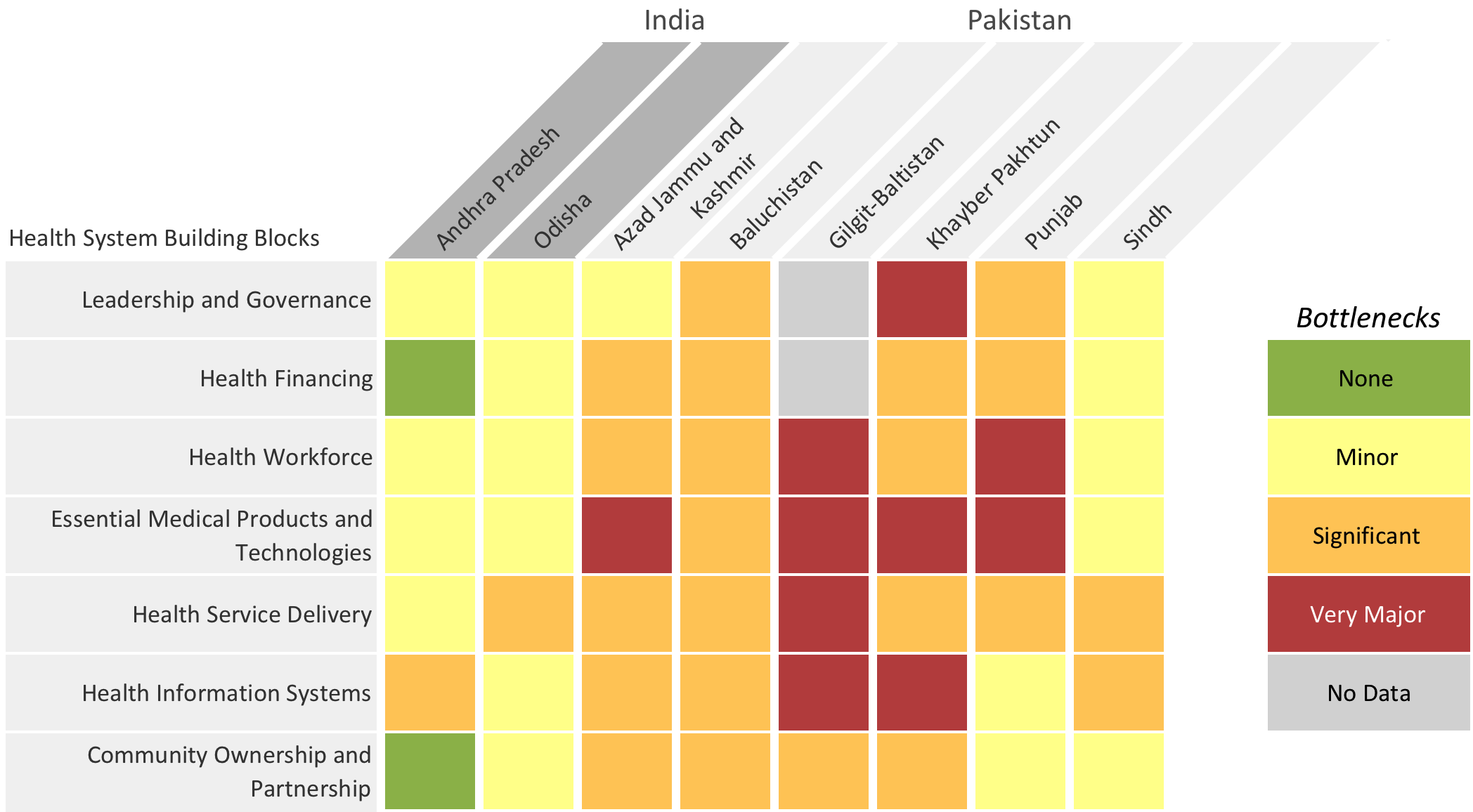


**BEmOC**


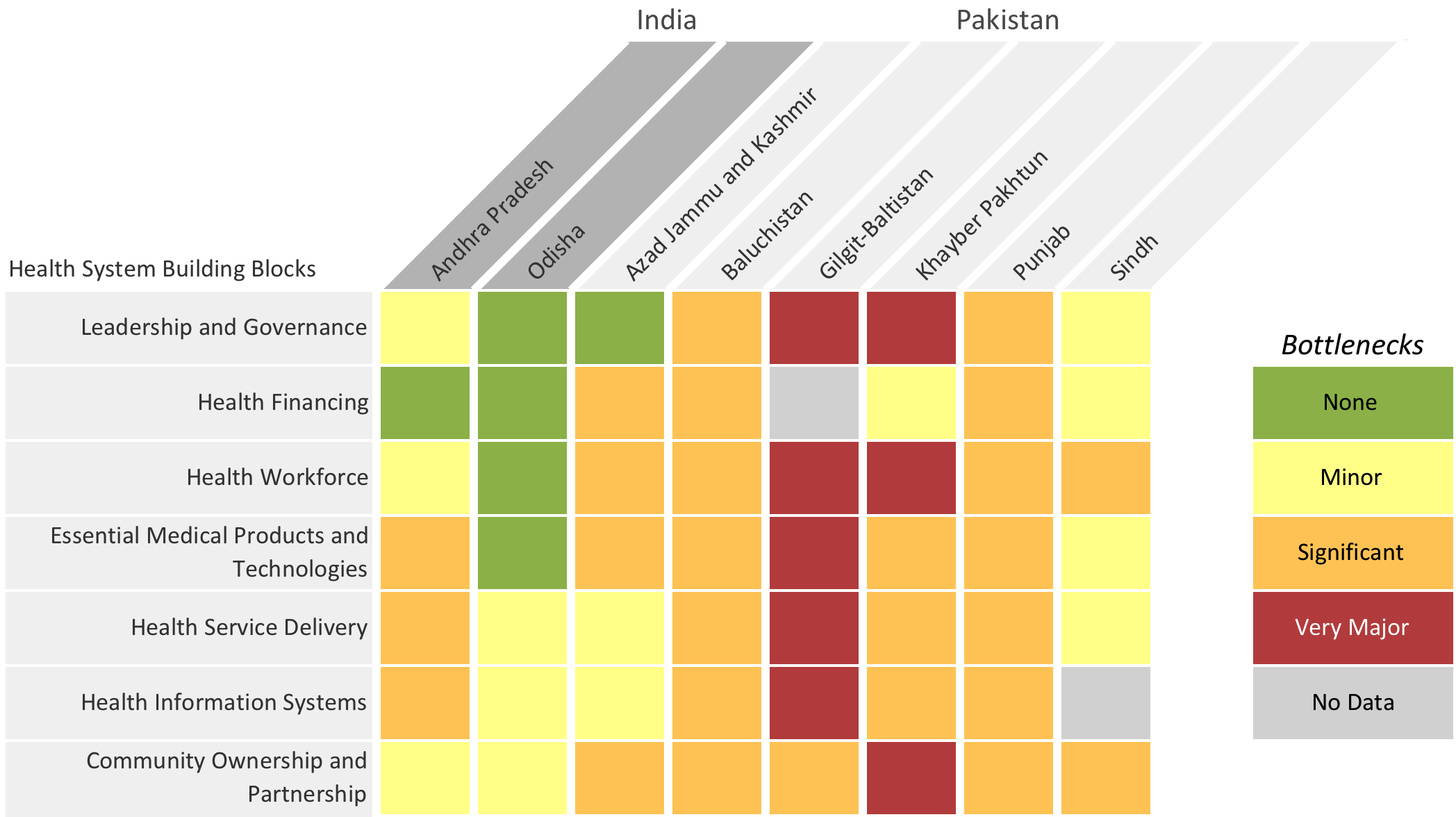


**CEmOC**


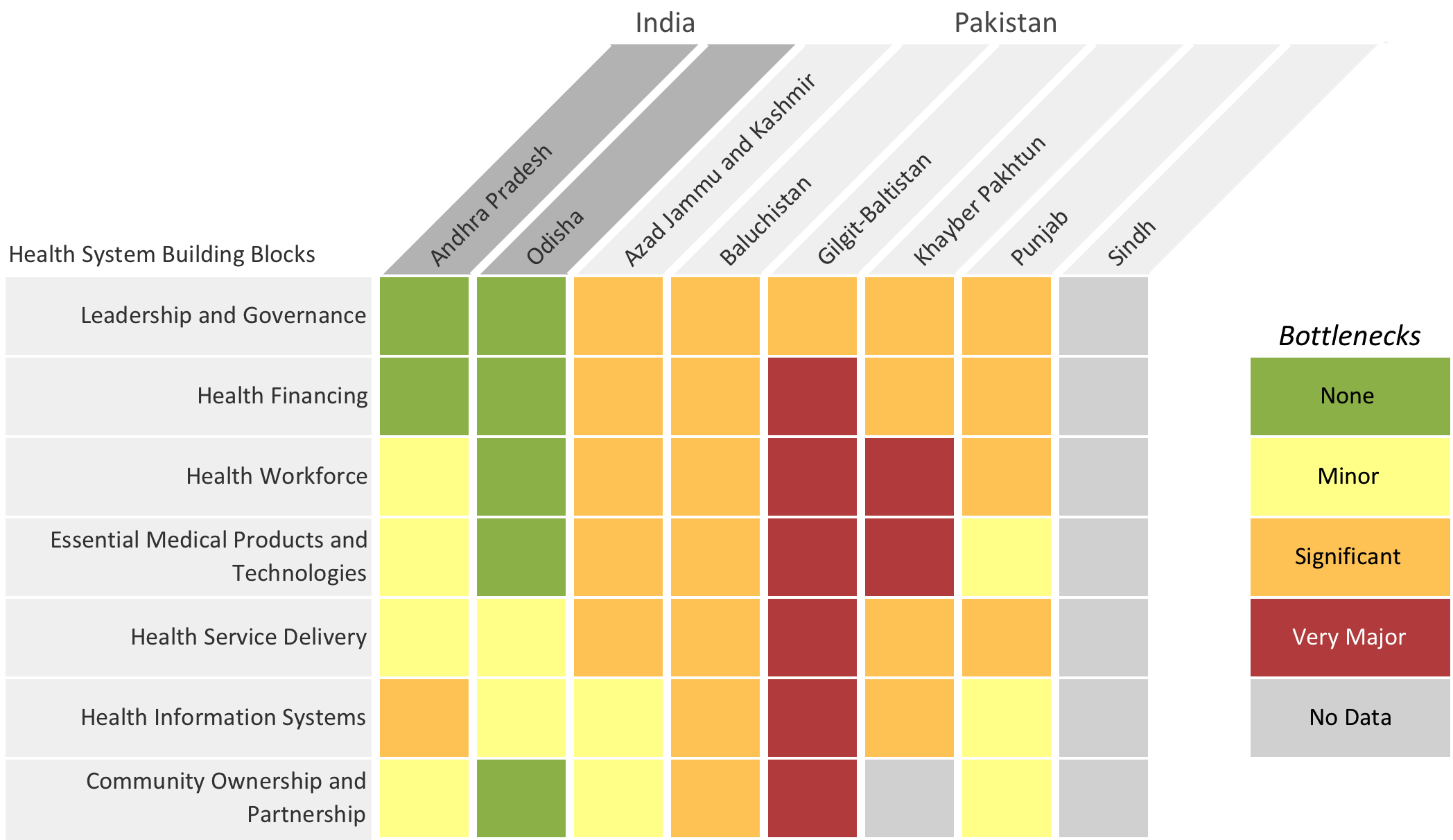


# J. Literature search strategy

**For the background section, we used the latest WHO and UN resources and used the following search terms in Pub Med. Limits were applied and only the relevant articles were retrieved.**

**Maternal newborn health or pregnancy related**

matern* OR pregnan* OR childbirth OR intrapartum OR intra-partum OR postpartum OR post-partum OR puerperal OR puerperium OR parturition OR expectant mother OR expectant mothers OR maternal health services OR delivery, obstetric OR parturition OR pregnancy OR Delivery, Obstetric OR postpartum period OR Obstetrics/ or Delivery, Obstetric/ or Pregnancy/ or Prenatal Care/ or Maternal Health Services/ or Infant, Newborn/ or neonat*or "Obstetrics and Gynecology Department, Hospital"/ or Pregnancy Complications/ or Obstetrics/ or Delivery, Obstetric/ or Pregnancy/ or Prenatal Care/ or Maternal Health Services/

For the discussion section, we searched the following search terms in Pub Med and google scholar. Only relevant articles were retrieved

**Health financing**

(Health) AND (financial access OR financial barrier OR out-of-pocket payment OR user fees OR conditional cash transfers OR cash benefits OR performance based incentives OR voucher OR reimbursement of transport costs) OR Budget allocation OR Innovative funding OR Social health insurance OR Universal health insurance OR Community based insurance OR National health insurance

**Health workforce**

(Health worker OR staff) AND (pre-service training OR in-service training OR recruitment OR recognition of midwifery staff OR skilled birth attendant OR doctor OR nurse OR training OR performance incentive OR retention OR contracting out OR increase in availability OR skill mix OR remuneration OR salaries) OR community health workers OR task shifting OR skills based training OR competency based training

**Health service delivery**

"Delivery of Health Care"/ or delivery of health care, integrated/AND health personnel/ or allied health personnel/ or community health aides/ or nurses/ or pharmacists/ or physicians/ AND health services/ or community health services/ or child care/ or infant care/ or intensive care, neonatal/ or perinatal care/ or child health services/ or exp maternal health services/ or immunization programs/ or mass vaccination/ or vaccination/ or rural health services/ AND quality assurance, health care/

# K. References

1. Black RE, Victora CG, Walker SP, Bhutta ZA, Christian P, de Onis M, *et al.*: **Maternal and child undernutrition and overweight in low-income and middle-income countries**. *Lancet* 2013, **382**(9890):427-451.

2. Secretary-General of the United Nations: **Global strategy for women’s and children’s health**. New York: United Nations; 2010.

3. World Health Organization; UNICEF: **Trends in maternal mortality: 1990 to 2013: estimates by WHO, UNICEF, UNFPA, The World Bank and the United Nations Population Division: executive summary**. 2014.

4. UNICEF; WHO; The World Bank; United Nations: **Levels and trends in child mortality: Report 2013.** 2013.

5. Lawn JE, Blencowe H, Pattinson R, Cousens S, Kumar R, Ibiebele I, *et al*.: **Stillbirths: Where? When? Why? How to make the data count?** *Lancet* 2011, **377**(9775):1448-1463.

6. Ronsmans C, Graham WJ: **Maternal mortality: who, when, where, and why**. *Lancet* 2006, **368**(9542):1189-1200.

7. Bang AT, Bang RA, Baitule SB, Reddy MH, Deshmukh MD: **Effect of home-based neonatal care and management of sepsis on neonatal mortality: field trial in rural India**. *Lancet* 1999, **354**(9194):1955-1961.

8. Baqui AH, El-Arifeen S, Darmstadt GL, Ahmed S, Williams EK, Seraji HR, *et al.*: **Effect of community-based newborn-care intervention package implemented through two service-delivery strategies in Sylhet district, Bangladesh: a cluster-randomised controlled trial**. *Lancet* 2008, **371**(9628):1936-1944.

9. Lawn JE, Mwansa-Kambafwile J, Horta BL, Barros FC, Cousens S: **‘Kangaroo mother care’to prevent neonatal deaths due to preterm birth complications**. *International journal of epidemiology* 2010, **39** Suppl 1:i144-i154.

10. Msemo G, Massawe A, Mmbando D, Rusibamayila N, Manji K, Kidanto HL, *et al.*: **Newborn mortality and fresh stillbirth rates in Tanzania after helping babies breathe training**. *Pediatrics* 2013, **131**(2):e353-360.

11. Prost A, Colbourn T, Seward N, Azad K, Coomarasamy A, Copas A, *et al.*: **Women's groups practising participatory learning and action to improve maternal and newborn health in low-resource settings: a systematic review and meta-analysis**. *Lancet* 2013, **381**(9879):1736-1746.

12. World Health Organization: **World Health Report 2005: Make every mother and child count. 2005**. *Geneva,* 2005.

13. Lawn JE, Lee AC, Kinney M, Sibley L, Carlo WA, Paul VK, *et al.*: **Two million intrapartum-related stillbirths and neonatal deaths: where, why, and what can be done?** *International Journal of Gynecology & Obstetrics* 2009, **107**:S5-S19.

14. Starrs AM: **Survival convergence: bringing maternal and newborn health together for 2015 and beyond**. *Lancet* 2014, **384**(9939):211-213.

15. WHO Essential Interventions: **Commodities and Guidelines for Reproductive, Maternal, Newborn and Child Health: A global review of the key interventions related to reproductive, maternal, newborn and child Health**. *Geneva: WHO,* 2011.

16. World Health Organization; UNICEF: **Monitoring emergency obstetric care: a handbook**. World Health Organization, 2009.

17. Kinney ML, Simen-Kapeu A, Moxon S, Kerber K, Matthai M, Powell-Jackson T, *et al*.: **PLACEHOLDER REFERENCE: Cross cutting health system bottlenecks and strategies to accelerate quality maternal and newborn care**. *BMC Pregnancy Childbirth* DRAFT.

18. World Health Organization, Department of Maternal Child and Adolescent Health: **Global Maternal, Newborn Child and Adolescent Health Policy Indicator Survey.** 2013.

19. Dickson KE, Simen-Kapeu A, Kinney MV, Huicho L, Vesel L, Lackritz E, *et al.*: **Every Newborn: health-systems bottlenecks and strategies to accelerate scale-up in countries**. *Lancet* 2014, **384**(9941):438-454.

20. Bustreo F, Say L, Koblinsky M, Pullum TW, Temmerman M, Pablos-Mendez A: **Ending preventable maternal deaths: the time is now**. *Lancet Global Health* 2013, **1**(4):E176-E177.

21. Ranson MK: **Reduction of catastrophic health care expenditures by a community-based health insurance scheme in Gujarat, India: current experiences and challenges**. *Bull World Health Organ* 2002, **80**(8):613-621.

22. Stenberg K, Axelson H, Sheehan P, Anderson I, Gulmezoglu AM, Temmerman M, *et al.*: **Advancing social and economic development by investing in women's and children's health: a new Global Investment Framework**. *Lancet* 2014, **383**(9925):1333-1354.

23. Lee AC, Lawn JE, Cousens S, Kumar V, Osrin D, Bhutta ZA, *et al.*: **Linking families and facilities for care at birth: what works to avert intrapartum-related deaths?** *International Journal of Gynecology & Obstetrics* 2009, **107**:S65-S88.

24. Witter S, Kusi A, Aikins M: **Working practices and incomes of health workers: evidence from an evaluation of a delivery fee exemption scheme in Ghana**. *Human resources for health* 2007, **5**(1):2.

25. Mohanty SK, Srivastava A: **Out-of-pocket expenditure on institutional delivery in India**. *Health Policy Plan* 2013, **28**(3):247-262.

26. Witter S: **Mapping user fees for health care in high-mortality countries–evidence from a recent survey**. In: HLSP Institute, London. 2010. http://eresearch.qmu.ac.uk/3026/1/Witter.pdf

27. El-Khoury M, Gandaho T, Arur A, Keita B, Nichols L: **Improving Access to Life Saving Maternal Health Services: The Effects of Removing User Fees for Caesareans in Mali**. *Bethesda: Health Systems* 2011, **20**:20.

28. Witter S, Dieng T, Mbengue D, Moreira I, De Brouwere V: **The national free delivery and caesarean policy in Senegal: evaluating process and outcomes**. *Health Policy and Planning* 2010, **25**(5):czq013.

29. Meessen B, Hercot D, Noirhomme M, Ridde V, Tibouti A, Bicaba A, *et al.*: **Removing user fees in the health sector in low-income countries: a multi-country review**. *New York: United Nations Children's Fund (UNICEF)* 2009:61-67.

30. Witter S, Khadka S, Nath H, Tiwari S: **The national free delivery policy in Nepal: early evidence of its effects on health facilities**. *Health policy and planning* 2011, **26**(suppl 2):ii84-ii91.

31. McPake B: **User charges for health services in developing countries: a review of the economic literature**. *Social science & medicine (1982)* 1993, **36**(11):1397-1405.

32. Gilson L, McIntyre D: **Removing user fees for primary care in Africa: the need for careful action**. *BMJ (Clinical research ed)* 2005, **331**(7519):762-765.

33. Witter S, Arhinful DK, Kusi A, Zakariah-Akoto S: **The experience of Ghana in implementing a user fee exemption policy to provide free delivery care**. *Reproductive health matters* 2007, **15**(30):61-71.

34. Burnham GM, Pariyo G, Galiwango E, Wabwire-Mangen F: **Discontinuation of cost sharing in Uganda**. *Bull World Health Organ* 2004, **82**(3):187-195.

35. Ridde V, Morestin F: **A scoping review of the literature on the abolition of user fees in health care services in Africa**. *Health Policy Plan* 2011, **26**(1):1-11.

36. Jehan K, Sidney K, Smith H, de Costa A: **Improving access to maternity services: an overview of cash transfer and voucher schemes in South Asia**. *Reproductive health matters* 2012, **20**(39):142-154.

37. Meng Q, Yuan B, Jia L, Wang J, Yu B, Gao J, Garner P: **Expanding health insurance coverage in vulnerable groups: a systematic review of options**. *Health Policy Plan* 2011, **26**(2):93-104.

38. Bellows NM, Bellows BW, Warren C: **Systematic Review: The use of vouchers for reproductive health services in developing countries: systematic review**. *Tropical Medicine & International Health* 2011, **16**(1):84-96.

39. World Health Organization; PMNCH: **PMNCH Knowledge Summary #21 Strengthen National Financing.** 2012.

40. Soeters R, Habineza C, Peerenboom PB: **Performance-based financing and changing the district health system: experience from Rwanda**. *Bull World Health Organ* 2006, **84**(11):884-889.

41. Spaan E, Mathijssen J, Tromp N, McBain F, ten Have A, Baltussen R: **The impact of health insurance in Africa and Asia: a systematic review**. *Bull World Health Organ* 2012, **90**(9):685-692.

42. Lim SS, Dandona L, Hoisington JA, James SL, Hogan MC, Gakidou E: **India's Janani Suraksha Yojana, a conditional cash transfer programme to increase births in health facilities: an impact evaluation**. *The Lancet* 2010, **375**(9730):2009-2023.

43. Rawlings LB, Rubio GM: **Evaluating the impact of conditional cash transfer programs**. *The World Bank Research Observer* 2005, **20**(1):29-55.

44. Anand S, Barnighausen T: **Human resources and health outcomes: cross-country econometric study**. *Lancet* 2004, **364**(9445):1603-1609.

45. World Health Organization: **Global Atlas of the Health Workforce** *online database, August 2009 update* 2009.

46. Gupta N, Maliqi B, Franca A, Nyonator F, Pate MA, Sanders D, *et al.*: **Human resources for maternal, newborn and child health: from measurement and planning to performance for improved health outcomes**. *Hum Resour Health* 2011, **9**(1):16.

47. Lehmann U, Dieleman M, Martineau T: **Staffing remote rural areas in middle- and low-income countries: a literature review of attraction and retention**. *BMC Health Serv Res* 2008, **8**(1):19.

48. Kirigia JM, Gbary AR, Muthuri LK, Nyoni J, Seddoh A: **The cost of health professionals' brain drain in Kenya**. *BMC Health Serv Res* 2006, **6**:89.

49. Ferrinho P, Van Lerberghe W, da Cruz Gomes A: **Public and private practice: a balancing act for health staff**. *Bull World Health Organ* 1999, **77**(3):209.

50. Fulton BD, Scheffler RM, Sparkes SP, Auh EY, Vujicic M, Soucat A: **Health workforce skill mix and task shifting in low income countries: a review of recent evidence**. *Hum Resour Health* 2011, **9**(1):1.

51. Sharma G: **Maternal, perinatal and neonatal mortality in South-East Asia Region**. *Asian Journal of Epidemiology* 2012, **5**(1):1-14.

52. McPake B, Mensah K: **Task shifting in health care in resource-poor countries**. *Lancet* 2008, **372**(9642):870-871.

53. Fenton PM, Whitty CJ, Reynolds F: **Caesarean section in Malawi: prospective study of early maternal and perinatal mortality**. *BMJ (Clinical research ed)* 2003, **327**(7415):587.

54. Kruk ME, Pereira C, Vaz F, Bergstrom S, Galea S: **Economic evaluation of surgically trained assistant medical officers in performing major obstetric surgery in Mozambique**. *BJOG: an international journal of obstetrics and gynaecology* 2007, **114**(10):1253-1260.

55. World Health Organization: **WHO recommendations: optimizing health worker roles to improve access to key maternal and newborn health interventions through task shifting**: World Health Organization; 2012.

56. Gabrysch S, Simushi V, Campbell OM: **Availability and distribution of, and geographic access to emergency obstetric care in Zambia**. *International journal of gynaecology and obstetrics* 2011, **114**(2):174-179.

57. Lumbiganon P, Laopaiboon M, Gulmezoglu AM, Souza JP, Taneepanichskul S, Ruyan P, *et al.*: **Method of delivery and pregnancy outcomes in Asia: the WHO global survey on maternal and perinatal health 2007-08**. *Lancet* 2010, **375**(9713):490-499.

58. Shah A, Fawole B, M'Imunya JM, Amokrane F, Nafiou I, Wolomby JJ, *et al.*: **Cesarean delivery outcomes from the WHO global survey on maternal and perinatal health in Africa**. *International journal of gynaecology and obstetrics* 2009, **107**(3):191-197.

59. Bullough C, Meda N, Makowiecka K, Ronsmans C, Achadi EL, Hussein J: **REVIEW: Current strategies for the reduction of maternal mortality**. *BJOG: An International Journal of Obstetrics & Gynaecology* 2005, **112**(9):1180-1188.

60. National Institute for Clinical Excellence: **Intrapartum care. Care of healthy women and their babies during childbirth**. *Clinical Guideline* 2007, **6**.

61. Austin A, Langer A, Salam RA, Lassi ZS, Das JK, Bhutta ZA: **Approaches to improve the quality of maternal and newborn health care: an overview of the evidence**. *Reproductive health* 2014, **11 Suppl 2**(Suppl 2):S1.

62. Donabedian A: **The quality of care: How can it be assessed?** *Jama* 1988, **260**(12):1743-1748.

63. Hulton L, Matthews Z, Stones RW: **A framework for the evaluation of quality of care in maternity services**. 2000.

64. Institute of Medicine; Committee on Quality of Health Care in America: **Crossing the quality chasm: A new health system for the 21st century.** National Academies Press; 2001.

65. Roemer; Montoya-Aguilar; World Health Organization: **Quality assessment and assurance in primary health care**. 1988.

66. World Health Organization: **Quality of care: a process for making strategic choices in health systems**. 2006.

67. van den Broek NR, Graham WJ: **Quality of care for maternal and newborn health: the neglected agenda**. *BJOG* 2009, **116 Suppl 1**(no. s1 ):18-21.

68. Sandin-Bojö A-K, Kvist LJ: **Care in Labor: A Swedish Survey Using the Bologna Score**. *Birth* 2008, **35**(4):321-328.

69. Raven J, Hofman J, Adegoke A, van den Broek N: **Methodology and tools for quality improvement in maternal and newborn health care**. *International journal of gynaecology and obstetrics* 2011, **114**(1):4-9.

70. Kruk ME, Mbaruku G, McCord CW, Moran M, Rockers PC, Galea S: **Bypassing primary care facilities for childbirth: a population-based study in rural Tanzania**. *Health Policy Plan* 2009, **24**(4):279-288.

71. Hanson K, Gilson L, Goodman C, Mills A, Smith R, Feachem R, *et al.*: **Is private health care the answer to the health problems of the world's poor?** *PLoS Medicine* 2008, **5**(11):e233.

72. Pomeroy AM, Koblinsky M, Alva S: **Who gives birth in private facilities in Asia? A look at six countries**. *Health policy and planning* 2014, **29**(suppl 1):i38-i47.

73. Madhavan S, Bishai D, Stanton C, Harding A: **Engaging the private sector in maternal and neonatal health in low and middle income countries**: Future health systems (FHS); 2010.

74. Bhat R, Mavalankar DV, Singh PV, Singh N: **Maternal healthcare financing: Gujarat's Chiranjeevi Scheme and its beneficiaries**. *Journal of health, population, and nutrition* 2009, **27**(2):249-258.

75. Singh A, Mavalankar DV, Bhat R, Desai A, Patel SR, Singh PV, *et al.*: **Providing skilled birth attendants and emergency obstetric care to the poor through partnership with private sector obstetricians in Gujarat, India**. *Bull World Health Organ* 2009, **87**(12):960-964.

76. Making Pregnancy Safer: **Making pregnancy safer: the critical role of the skilled attendant**. 2004.

77. Ministry of Health; Government of Malaysia: **Health facts 2012**. 2012.

78. Ravichandran J, Ravindran J: **Lessons from the confidential enquiry into maternal deaths, Malaysia**. *BJOG* 2014, **121 Suppl 4**(s4):47-52.

79. Government of Malaysia: **Reports on the Confidential Enquiries into Maternal Deaths in Malaysia 2009–2012.** 2012.

80. Ravindran J: **Management of the adherent placenta-practice considerations.** *J Paediatr Obstet Gynaecol* 2013, **39:93-9.**

81. Ravindran J, Shamsuddin K, Selvaraju S: **Did we do it right?-An evaluation of the colour coding system for antenatal care in Malaysia**. *Medical Journal of Malaysia* 2003, **58**(1):37-53.

82. Karim R, Ali SH: **Maternal health in Malaysia: progress and potential**. *Lancet* 2013, **381**(9879):1690-1691.
